# Strength of Interlayer Metal–Metal Coupling as Key Active Site Configuration and Atomic Descriptor for Single-Atom Catalysts

**DOI:** 10.1021/jacs.6c04989

**Published:** 2026-07-09

**Authors:** Liangliang Xu, Jiankang Wang, Hanxu Yao, Jinpei Huang, Zijing Li, Xingkun Wang, Linguo Lu, Jian Zhou, Peixin Cui, Heqing Jiang, Zhengxiao Guo

**Affiliations:** † Department of Chemistry, 25809 The University of Hong Kong, Hong Kong SAR 999077, China; ‡ State Key Laboratory of Photoelectric Conversion and Utilization of Solar Energy, Qingdao New Energy Shandong Laboratory, Qingdao Institute of Bioenergy and Bioprocess Technology, Chinese Academy of Sciences, Qingdao 266100, P. R. China; § Graduate School of Advanced Imaging Science, 26729 Chung-Ang University, Seoul 06974, South Korea; ∥ Department of Chemistry, University of Puerto Rico, Rio Piedras, San Juan, Puerto Rico 00931, United States; ⊥ Physical Chemistry, University of Konstanz, Universitätsstraße 10, Box 714, Konstanz 78457, Germany; # State Key Laboratory of Soil & Sustainable Agriculture, Institute of Soil Science, Chinese Academy of Sciences, Nanjing 210008, China; ¶ Hong Kong Quantum AI Lab, AIR@InnoHK of Hong Kong Government, Hong Kong SAR 999077, China

## Abstract

Single-atom catalysts (SACs) are widely considered for large-scale applications due to their exceptional activity, selectivity, and near-unity atom economy, where active-site configuration is critical. Yet how the active site regulates catalytic performance remains incompletely understood. A long-standing example is layered Fe–N–C, which shows a large discrepancy between theoretical predictions and experimental oxygen reduction reaction (ORR) activity, limiting rational design and predictability. Here, guided by X-ray absorption fine structure (XAFS) observations and supported by simulations and machine learning, we identify a stacked bilayer metal–metal coupling (MMC) configuration as an important active-site motif in these catalysts. Without invoking constant-potential treatments or surface hydroxyl coverage, our model, constructed within the standard computational hydrogen electrode (CHE) framework, reproduces the experimental activity and reconciles theory with experiment, supporting MMC as a plausible and important mechanistic contributor to ORR. By constructing and comparing 15 single-layer (SL) and MMC configurations, we quantify how metal–metal coupling strength governs activity and establish a criterion for selecting appropriate modeling strategies based on coupling intensity. Using machine learning and data mining, we further identify local electronic-structure descriptors that enable quantitative structure–activity relationships to guide catalyst design. Finally, we synthesize a series of molecular catalysts featuring bilayer MMC motifs or isolated single-metal sites, thereby experimentally validating the proposed structural motif and its role in ORR characteristics. This discovery provides guidance for the rational design of SACs and highlights that physically faithful active-site modeling is a prerequisite for predictive theory, with transferable implications for electrocatalytic systems beyond ORR.

## Graphical abstract

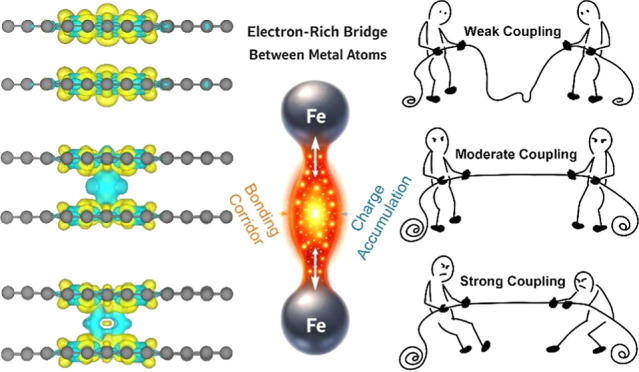

## Introduction

Oxygen reduction reaction (ORR) plays a pivotal role in energy conversion and storage, particularly in fuel cells and metal-air batteries, where the reaction kinetics largely determine the energy conversion efficiency and operational stability of the devices.^1–4^ However, due to its inherently complex multielectron transfer processes,^5,6^ achieving precise control over intermediate adsorption remains challenging, which in turn results in sluggish kinetics and elevated overpotentials in practice.^7–9^ Currently, platinum (Pt)-based catalysts are at the forefront of cathodic ORR in commercial hydrogen fuel cells, due to their exceptional catalytic activity and stability.^10,11^ Nevertheless, the high cost and limited availability of Pt significantly hinder its large-scale deployment in energy systems aiming for net-zero emissions. In recent years, increasing attention has been shifted toward metal–nitrogen-carbon (M–N–C) single-atom catalysts (SACs), which exhibit several advantages including low cost, high atomic dispersion, tunable coordination environments, and excellent electrochemical stability.^12–16^ Such features enable M–N–C catalysts to perform comparably with, or even superior to, noble metal catalysts.

Among those, iron–nitrogen-carbon (Fe–N–C) systems have emerged as one of the most promising nonprecious metal alternatives to Pt-based ORR catalysts.^14,17–19^ However, the fundamental mechanism by which Fe–N–C catalysts regulate ORR activity at the electronic structure level remains unclear, particularly given the significant discrepancy between the limiting potentials (η_ORR_ ≈ 0.25–0.43 V)^20–22^ predicted by the conventional computational hydrogen electrode (CHE) model^23,24^ and the experimentally inferred values from half-wave potentials, which typically differ by about 0.4 V.^14,15,25,26^ This issue has prompted a wide range of corrective strategies. Some studies have proposed a reconfiguration of the reaction pathway, such as modified *OOH formation or dissociation steps.^20^ Others suggest that under working electrochemical conditions, the active site may be covered by OH intermediates,^27^ thereby altering adsorption behavior. It has also been proposed that the modification of axial ligands on active metal centers is an efficient and universal strategy for tuning the activity of single-atom catalysts.^28^ In addition, it has been argued that the CHE framework does not explicitly account for the effect of applied electrode potential,^29^ and thus constant-potential approaches have been introduced to reproduce more accurately the experimental trends.^30–34^

Although these methods have bridged the theoretical-experimental gap for Fe–N–C systems to some extent, their applicability has largely been limited to Fe-centered configurations. In contrast, structurally analogous Co–N–C catalysts exhibit excellent agreement between CHE-based predictions and experimental data, with negligible deviations,^35^ and this discrepancy across different metal centers has yet to be systematically explained.^36^ Therefore, it remains an open question whether a generalized structural-electronic regulation mechanism exists that can be applied across a broader range of transition metal–nitrogen-carbon (TM–N–C) systems. Addressing this challenge not only provides a unified understanding of how mechanistic accuracy varies with metal identity, but also establishes a theoretical foundation for transferable modeling strategies and universal structure–activity relationships (SARs) in SACs, thereby filling a critical gap in current mechanistic and methodological frameworks.

Based on the above analysis, we seek to investigate a possible structural origin of the substantial deviation between theoretical predictions and experimental observations in Fe–N–C systems for ORR, based on the *K*-edge extended X-ray absorption fine structure (EXAFS) characterization, density functional theory (DFT) calculations and machine-learning (ML).^37^ We attribute the long-standing theory-experiment deviation primarily to an incomplete identification of the catalytically relevant active site. Because catalytic efficiency and selectivity are ultimately determined by the local electronic structure of the true operating site, establishing an experimentally grounded site model is a prerequisite for predictive electrocatalysis. Notably, Xie et al. report an additional shoulder-like feature in the Fe K-edge FT-EXAFS that was assigned to an Fe–Co scattering contribution, providing direct spectroscopic evidence for metal–metal interaction in a face-to-face coupled FeN_4_/CoN_4_ bilayer motif.^38^ Similarly, Lv et al. report that coupling FePc with atomic Fe–N_4_ sites introduces a new shoulder peak in the Fe K-edge X-ray absorption near-edge structure (XANES), and EXAFS fitting requires a higher-shell Fe–Fe contribution, consistent with intersite metal–metal coupling that perturbs the local Fe–N_4_ electronic structure.^39^ Motivated by the above observations, we propose a stacked bilayer metal–metal coupling (MMC) configuration to account for such interactions, aiming to address possible limitations of conventional single-atom models and significantly improve the accuracy of ORR limiting potential predictions, as well as subsequent design of mechanistic pathways. We further extend this approach to other TM–N–C systems. Computational results demonstrate that the predicted limiting potential (0.95 V) of the (Fe–Fe)­N_4_ configuration is in excellent agreement with the experimental values reported to date, including a literature half-wave potential of ∼0.90 V^26^ and our own experimental value of 0.88 V. In contrast, for systems with intrinsically weak coupling (e.g., CoN_4_ and (Co–Co)­N_4_), both the single-layer (SL) model and MMC yield similar results that align well with experiments, further confirming the selective applicability of the MMC.

Building on this framework, we establish a model selection criterion based on the strength of metal–metal coupling, enabling rational identification of systems that require metal–metal cooperative structures for accurate prediction. Furthermore, through data mining analysis, we identify a set of electronic descriptors that quantitatively capture the SAR of the mixed SL + MMC in relation to catalytic performance. Importantly, the MMC framework is further validated through a carefully designed experimental system featuring a bilayer-like architecture, which confirms the model’s transferability and predictive power at both the structural and performance levels. This finding not only provides new insight into viable active-site configurations in SACs but also offers a theoretical foundation and practical strategy for the unified modeling and rational design of TM–N–C catalysts across different metal centers (Figure 1).

**Figure 1:**
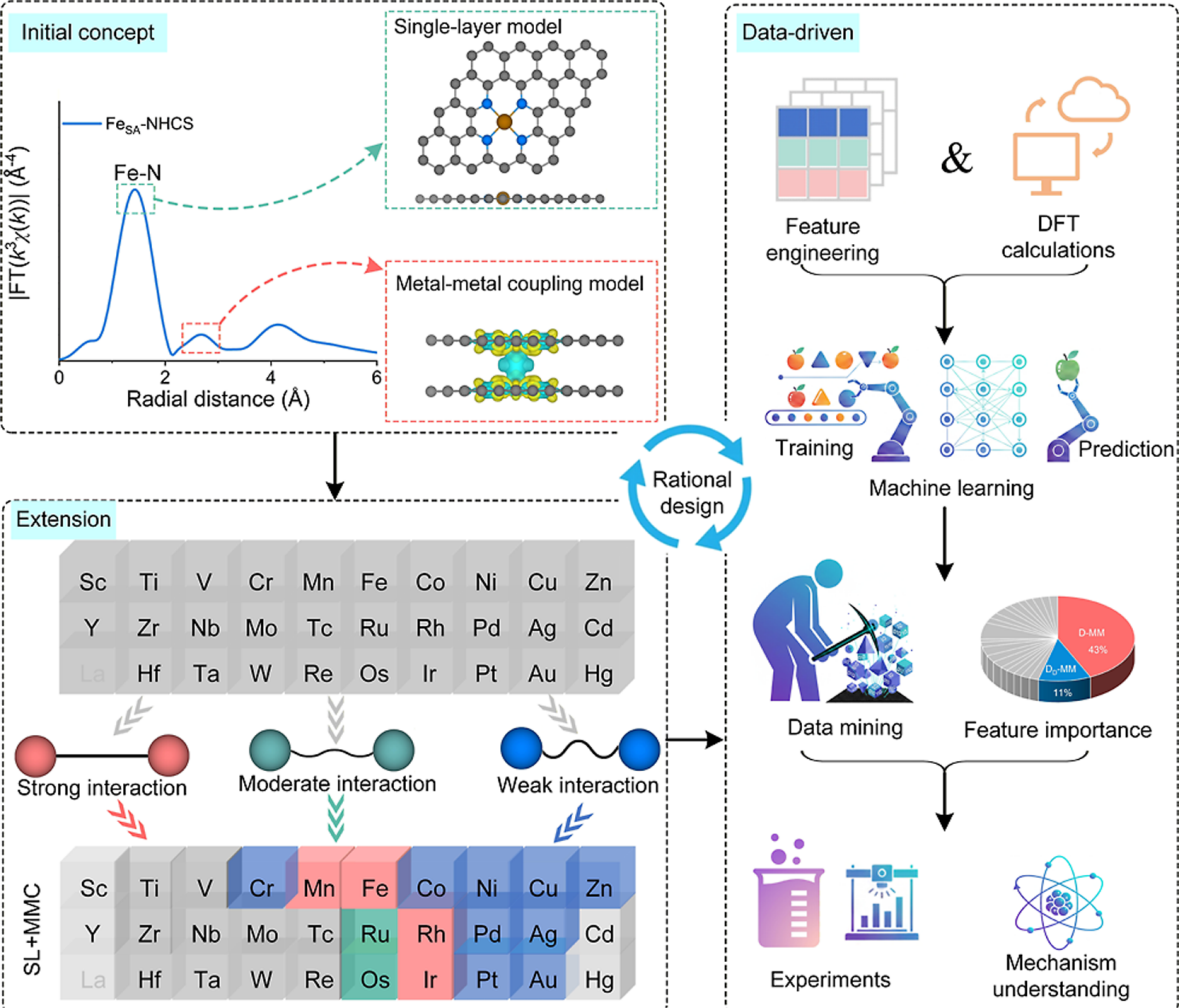
M–M coupling-inclusive modeling and data-driven design of transition metal–nitrogen-carbon (TM–N–C) single-atom catalysts (SACs).

## Results

### EXAFS-Guided Construction of a Bilayer Metal–Metal Coupling Model

To investigate the origin of the theoretical-experimental deviation in Fe–N–C systems, we first proposed the hypothesis of Fe–Fe coupling based on extended X-ray absorption fine structure (EXAFS) characterization. Specifically, the appearance of a subtle additional feature adjacent to the Fe–N coordination shell suggests that the Fe center may weakly couple with a nearby Fe atom, introducing metal–metal interactions that are not captured by conventional single-site models and thereby contributing to the long-standing discrepancy between computed and experimentally observed ORR activity (Table S1). Guided by this hypothesis, we constructed a double-layer MMC model. Prior to establishing the MMC, we systematically evaluated various stacking configurations between the upper and lower Fe sites. Using the Fe–N–C system as a representative case, we calculated the relative energies of different Fe–Fe distances, as shown in Figure S1. The configuration with vertically aligned Fe atoms exhibits the lowest energy, indicating the most thermodynamically favorable structure. To further examine whether interlayer C–C repulsion could induce lateral dislocation between the two graphitic layers, we additionally considered several laterally shifted bilayer registries for the Fe–Fe MMC model (Figure S2). The aligned configuration used in the main text remains the lowest-energy structure among the cases examined, whereas the laterally shifted registries are higher in energy by 0.17, 0.34, and 0.37 eV, respectively. These results indicate that although interlayer dislocation is in principle possible in graphitic systems, the coupled Fe–Fe motif in the aligned MMC configuration is energetically favored, while larger lateral offsets are distinctly less stable.

To further verify the structural optimality of the vertical alignment, we varied the initial Fe–Fe distance from 2.0 to 3.6 Å and performed geometry optimizations. As shown in Figure 2a, the optimized structures fall into two main categories with distances around 2.40 and 2.75 Å, respectively. The configuration with ∼2.75 Å exhibits lower energy and matches well with the stable configuration in Figure S1 (2.78 Å), thus validating the MMC geometry.

**Figure 2:**
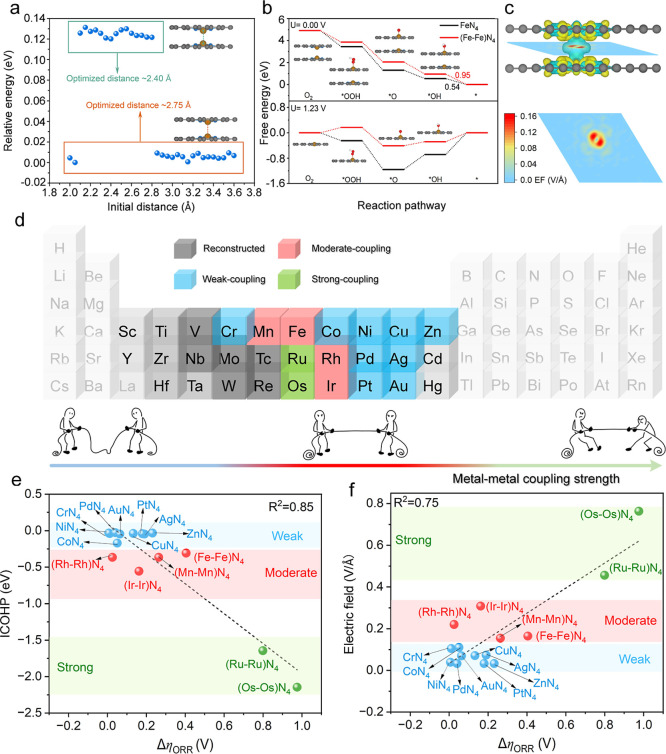
(a) Relative energies of FeN_4_-MMC configurations after optimization from different initial Fe–Fe distances. Inset shows the optimized structure. (b) Free energy diagram of FeN_4_–SL and (Fe–Fe)­N_4_-MMC configurations. (c) Charge density difference and electric field intensity distribution at the coupling interface of the (Fe–Fe)­N_4_-MMC. Yellow and cyan denote charge accumulation and depletion, respectively. The isosurface value is set to 0.003 e Å^–3^. (d) Twenty-nine transition metal MMC configurations. (e) Correlation between the difference in predicted ORR limiting potential (Δη_ORR_) by MMC and SL with the calculated integrated crystal orbital Hamilton population (ICOHP) values. (f) Correlation between the Δη_ORR_ and the electric field.

Subsequently, we calculated the ORR free energy profiles. As illustrated in Figure 2b, the traditional single-layer FeN_4_ model yields a limiting potential of 0.54 V at *U* = 0 V, which significantly underestimates the experimental value (∼0.9 V), highlighting the limitations of the conventional model. In contrast, the bilayer MMC configuration (Fe–Fe)­N_4_ achieves a theoretical limiting potential of 0.95 V, which closely agrees with experimental data. To exclude possible methodological effects, we further performed comparative calculations by considering DFT + U correction and solvation effects, as shown in Figure S3. The results indicate that DFT + U correction and solvation treatment are not the primary factors responsible for the activity differences among these systems. This strongly suggests that the metal–metal coupling is an important contributing factor affecting ORR activity.

To rule out the possibility that the activity enhancement arises from weak interactions in adjacent Fe sites within a SL model, we designed two single-layer (Fe–Fe)­N_4_–SL configurations with different Fe–Fe distances (3.17 Å and 4.99 Å, Figure S4). The computed limiting potentials for these models are 0.52 and 0.48 V, respectively, both of which still significantly deviate from experimental values (∼0.90 V). Therefore, weak interactions within single layers are insufficient to account for the activity enhancement, implying that another important Fe–Fe coupling configuration may be involved.

Since OH coverage has previously been proposed as a factor affecting ORR energetics in Fe–N–C systems,30 we further tested OH-ligated MMC models for representative Fe-, Co-, and Ni-based cases (Figure S5). For (Fe–Fe)­N_4_, OH ligation lowers the limiting potential from 0.95 to 0.67 V, thereby worsening the agreement with experiment, whereas only negligible changes are observed for (Co–Co)­N_4_ (0.80 to 0.80 V) and (Ni–Ni)­N_4_ (0.12 to 0.13 V). This comparison suggests that OH coverage is not a universally additive correction to MMC, but a motif- and system-dependent effect. In the Fe–Fe MMC case, the bare coupled motif already captures the dominant structural effect needed to reproduce the experimental trend, and additional OH ligation instead leads to overcorrection.

Attention was thus paid to M–M coupling between adjacent layers. As shown in Figure 2c, the charge density difference demonstrates significant charge redistribution between the two Fe atoms, forming an electron-rich “bridge” (or “bonding corridor”) that reflects strong metal–metal coupling across the layers. The associated electric field mapping reveals a concentrated field at the interface, highlighting a tip-enhancement effect. These results collectively indicate that Fe–Fe coupling in the MMC gives rise to substantial local electric field modulation and charge polarization, which play a critical role in tuning the adsorption behavior of key ORR intermediates and enhancing the catalytic performance.

To evaluate the universality of the proposed MMC across transition metal-based SACs, we systematically investigated a total of 29 transition metals spanning the 3d, 4d, and 5d blocks in the periodic table, as illustrated in Figure 2d. Among them, elements in the light gray region (such as Sc, Y, Cd, and Hg) tend to form high-coordination M-N_8_ structures due to their large atomic radii or electronic configuration constraints, resulting in strong M-N bonding but lacking effective M–M coupling. Elements in the medium gray region (Ti, Zr, Hf, Ta) induce layer sliding and structural reconstruction of the graphene substrate upon *O adsorption, leading to a change to M-N_6_ configurations, thereby deviating from the original structural paradigm. Meanwhile, elements in the dark gray zone (V, Nb, Mo, W, Ru, Re) exhibit spontaneous dissociation of *OOH intermediates, forming oxygen-bridged motifs distinct from the canonical adsorption mechanism of conventional SL (Figure S6), rendering them unsuitable for uniform comparative analysis.

After excluding these anomalous cases, we identified 15 representative transition metal systems that maintain comparable coordination environments, consistent reaction pathways (Figure S7), and structural stability. To further assess the electrochemical stability of the representative metal centers, we calculated the dissolution potentials of the Fe-, Co-, and Ni-based SL and MMC models. The dissolution potentials of FeN_4_–SL, CoN_4_–SL, and NiN_4_–SL are 0.77, 1.02, and 1.21 V, respectively, whereas those of (Fe–Fe)­N_4_-MMC, (Co–Co)­N_4_-MMC, and (Ni–Ni)­N_4_-MMC increase to 2.38, 2.66, and 2.82 V, respectively. The higher dissolution potentials indicate that the MMC models possess stronger resistance to metal dissolution than the corresponding SL models (The computational details are provided in the Supporting Information). For each system, the corresponding MMC was constructed and quantitatively evaluated based on the metal–metal coupling strength. Specifically, we calculated the integrated crystal orbital Hamilton population (ICOHP) values to assess M–M bonding strength and evaluated the electric field (EF) intensity in the interfacial electron-rich bridging region to characterize coupling-induced field effects (Figures S8 and S9). As shown in Figure 2d, the selected systems were classified into three coupling strength regimes: strong (green, e.g., Ru–Ru, Os–Os), moderate (red, e.g., Fe–Fe, Mn–Mn, Rh–Rh, Ir–Ir), and weak (blue, including Cr–Cr, Co–Co, Ni–Ni, Cu–Cu, Zn–Zn, Pd–Pd, Ag–Ag, Pt–Pt, and Au–Au).

To assess the correlation between coupling strength and catalytic prediction accuracy, we further analyzed the relationship between the difference in predicted ORR limiting potentials (Δη_ORR_) from MMC and SL and the computed ICOHP and EF values. As shown in Figure 2e, a strong negative correlation is observed between ICOHP and Δη_ORR_, with a fitting coefficient of *R* ^2^ = 0.85, indicating that stronger M–M bonding results in a greater difference in η_ORR_ between the two systems. Similarly, Figure 2f reveals a positive correlation between EF intensity and Δη_ORR_ (*R* ^2^ = 0.75), confirming the EF intensity as a reliable descriptor of coupling effects (The Bader charge does not appear to be a reliable descriptor for characterizing the coupling strength, as shown in Figures S10 and S11). In particular, weakly coupled systems (e.g., Ni–Ni, Co–Co) show negligible changes in limiting potential upon MMC implementation, suggesting that conventional single-layer models suffice for accurate performance prediction. In contrast, moderately (e.g., Fe–Fe) and strongly (e.g., Ru–Ru) coupled systems require MMC to reconcile theoretical predictions with experimental observations, highlighting the necessity of incorporating M–M interactions in such cases. Consistent with this picture, the binding energy also exhibits a similar correlation with Δη_ORR_ (Figure S12). To further distinguish the intrinsic metal–metal interaction from the influence of the surrounding coordination environment, we additionally calculated ICOHP values for isolated metal–metal dimers in vacuum. The resulting correlation with Δη_ORR_ remains significant (Figure S13), indicating that the intrinsic metal–metal interaction itself captures the dominant overall trend, while the surrounding framework further modulates the magnitude of the effect. These findings underscore the essential role of metal–metal coupling strength in accurate ORR activity prediction and offer a robust framework for model selection and descriptor development across diverse transition metal–N–C catalyst systems.

### Mapping the Mixed SL + MMC Activity Landscape: From Scaling Relations to Machine Learning

Although we have established a classification criterion between the SL and the MMC based on coupling strength, the dominant factors underlying the overall ORR activity of catalytic systems composed of both mixed models (SL + MMC) remain unclear. To further clarify this issue, we first analyzed the scaling relationship between adsorption free energies (Figure 3a) and the volcano-type relationship between adsorption free energy and limiting potential (η_ORR_) (Figure 3b). The fitted results in Figure 3a reveal strong linear correlations among intermediates, validating the interdependence between adsorption energies. The volcano plot in Figure 3b follows the Sabatier principle, indicating that optimal catalytic activity generally corresponds to moderate adsorption strength, neither too strong nor too weak. Notably, the (Fe–Fe)­N_4_ configuration lies near the apex of the volcano plot, exhibiting the best ORR performance and further supporting the rationality of the MMC model. To enhance the generality of our conclusions, we also evaluated the scaling and volcano relationships for all independent SL configurations (Figure S14) and all independent MMC configurations (Figure S15). The results showed good trend fitting for both subsets, indirectly validating the consistency between the classification strategy and the actual evolution of electronic structure, and confirming that the mixed SL + MMC systems possess a continuous and analyzable structure–performance mapping relationship.

**Figure 3:**
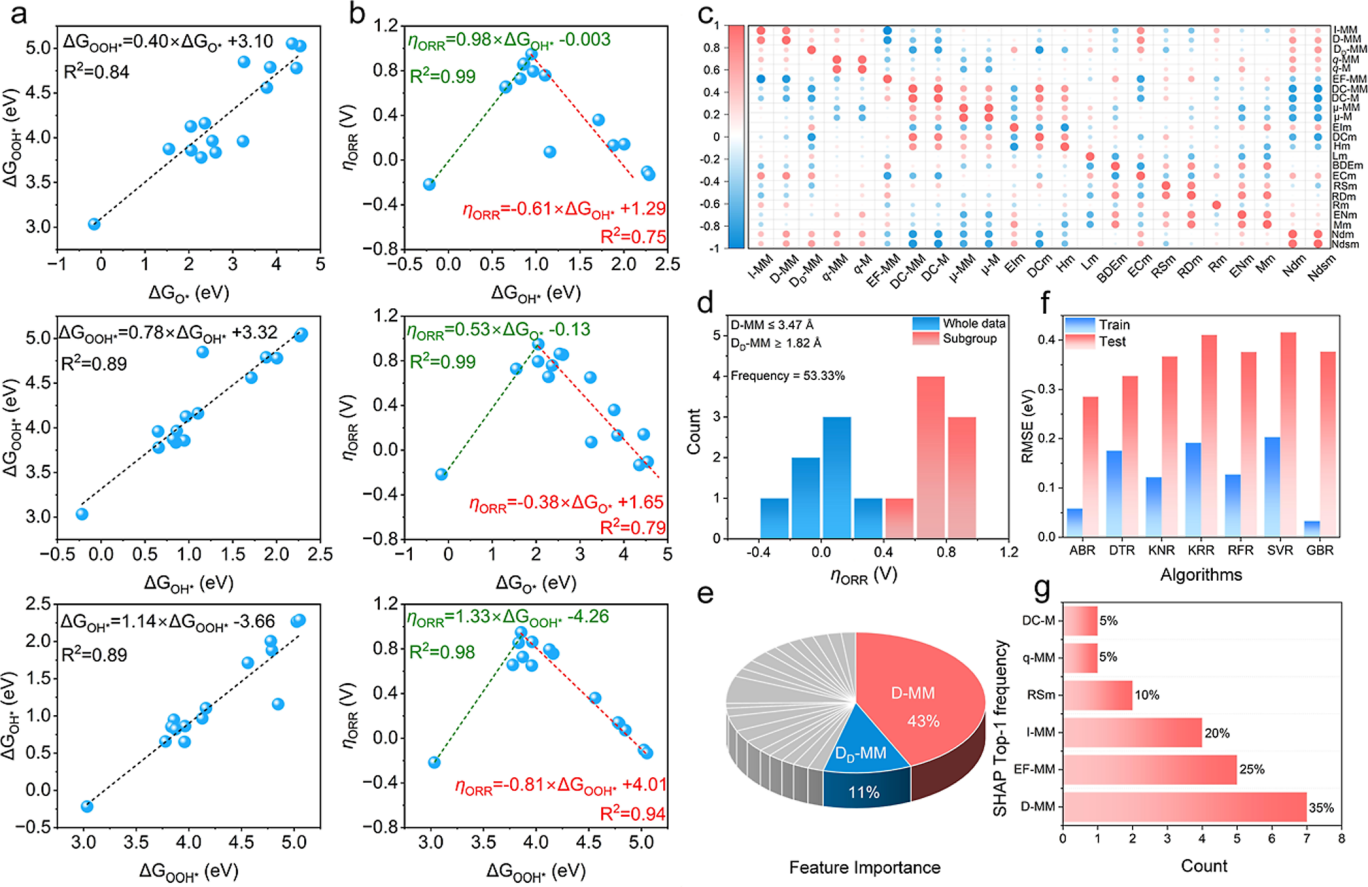
(a) Relationships among adsorbed intermediates in the mixed SL + MMC system. (b) Correlation between adsorption energetics of intermediates and η_ORR_. (c) Correlation heatmap among all features. (d) Subgroup data sets of η_ORR_ in the mixed SL + MMC system selected by the subgroup discovery. (e) Feature importance analysis. (f) Benchmarking of six regression models. (g) Resampling-based robustness of SHAP feature importance (Top-1 selection-frequency).

To further investigate which descriptors govern the ORR activity of the SL + MMC mixed systems, we employed the subgroup discovery (SGD) data mining method.^40–42^ The SGD approach is notably robust against data scarcity and can efficiently extract localized high-activity patterns under limited sample conditions. The correlation heatmap in Figure 3c covers a range of structural and electronic descriptors (see Table 1), reflecting the diversity in feature selection. As shown in Figure 3d, SGD identified two key descriptors: the postcoupling metal–metal distance D-MM ≤ 3.47 Å and the relaxed natural dimer distance D_D_-MM ≥ 1.82 Å. This subgroup covers approximately 53.33% of the entire data set. Figure 3e further reveals that these two descriptors dominate the feature importance ranking, where D-MM accounts for 43% and D_D_-MM for 11%, together exceeding half of the total contribution.

**Table 1:** Initial Features Defined for Machine Learning

categories	identified features	abbreviation
surface	integrated crystal orbital Hamilton population value of metal–metal for MMC (eV)	I-MM
	distance of metal–metal for MMC (Å)	D-MM
	dimer distance of metal (Å)	D_D_-MM
	average Bader charge of metal–metal for MMC (e^–^)	*q*-MM
	average Bader charge of metal–metal for SL (e^–^)	*q*-M
	electric field of MMC (eV)	EF-MM
	D-band center of MMC (eV)	DC-MM
	D-band center of SL (eV)	DC-M
	magnetic moment of MMC (μB)	μ-MM
	magnetic moment of SL (μB)	μ-M
atomic	first ionization energy of metal (eV)	EIm
	D-band center of metal (eV)	DCm
	highest occupied orbital of metal (eV)	Hm
	lowest occupied orbital of metal (eV)	Lm
	the dimer bond dissociation energy of metal (eV)	BDEm
	the bulk cohesive energy of metal (eV)	ECm
	radius of s-orbital of metal (Å)	RSm
	radius of d-orbital of metal (Å)	RDm
	atomic radius of metal (Å)	Rm
	Pauling electronegativity of metal	ENm
	relative atomic mass (a.m.u.) of metal	Mm
	number of d valence electrons of metal	Ndm
	total number of d and s valence electrons of metal	Ndsm

In parallel, to assess descriptor relevance from a global, model-based perspective, we compared six supervised learning algorithms (Figure 3f), among which gradient boosting regression (GBR) provides the best predictive performance. Given the limited sample size, we evaluated the stability of GBR-based feature attribution via repeated random train/test splits: the data set was randomly partitioned 20× (80% training, 20% testing), and SHAP analyses were performed for each split. The resulting Top-1 selection-frequency ranking (Figure 3g) consistently identifies D-MM as the most frequently selected leading descriptor, while the features most often appearing in the Top-3 set are summarized in Figure S16. Notably, EF-MM and I-MM follow closely behind D-MM, consistent with our earlier coupling-strength analysis based on electric-field intensity and ICOHP. This agreement between the local, rule-based SGD results and the global, resampling-stabilized SHAP attributions supports a coherent physical picture: geometric proximity (D-MM) acts as an effective gatekeeper for identifying coupling-relevant systems, whereas electronic descriptors such as EF-MM and I-MM capture the strength of metal–metal interaction and its impact on adsorption energetics across the data set. Collectively, these results indicate that high-performance SL + MMC mixed systems tend to exhibit strong metal–metal coupling (small D-MM), but such coupling should not originate from the intrinsic proximity of metal atoms (large D_D_-MM). Instead, it is associated with coupling-induced electronic reconfiguration and interfacial polarization, which jointly modulate the interaction between the active site and ORR intermediates. This finding not only clarifies the mechanistic origin of activity enhancement in mixed SL + MMC configurations, but also provides a quantitative and transferable criterion for catalyst screening and rational structural design.

### Electronic Structure Origin of Coupling-Enhanced ORR Activity

To gain an in-depth understanding of the intrinsic mechanism by which metal–metal coupling enhances catalytic activity, we systematically analyzed the electronic structures of selected systems with different coupling strengths: noncoupled (Ni–Ni), weakly coupled (Co–Co),^43^ moderately coupled (Fe–Fe),^14,15^ and strongly coupled (Ru–Ru),^44^ as shown in Figure 4a. All MMC structures were treated using spin-polarized DFT. The electronic structures discussed below correspond to the converged spin-polarized solutions obtained for each configuration under a consistent computational protocol, enabling a uniform comparison across the MMC data set.^45–49^

**Figure 4:**
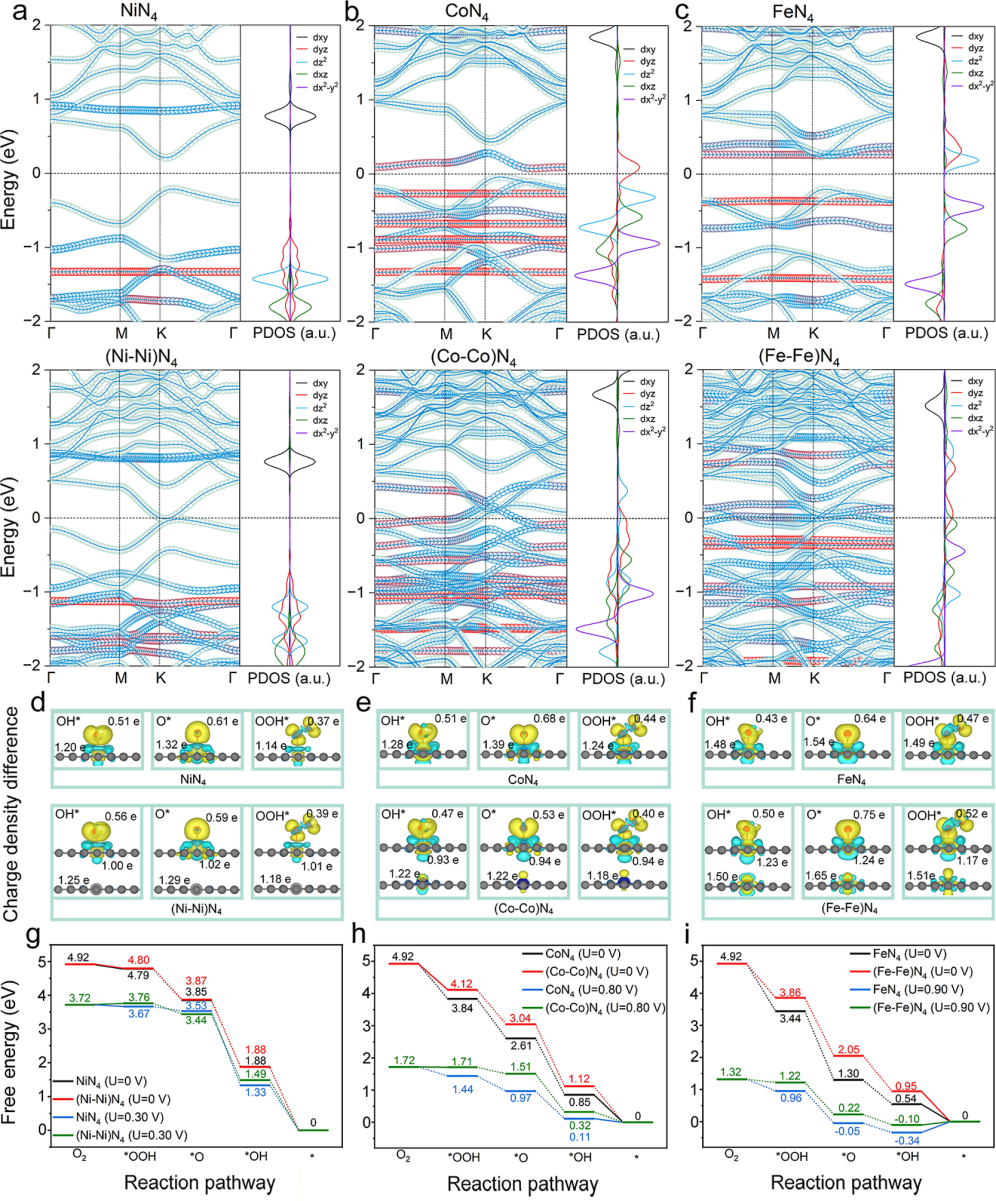
Projected band structures (PBS) and projected density of states (PDOS) of (a) NiN_4_, (Ni–Ni)­N_4_, (b) CoN_4_, (Co–Co)­N_4_, and (c) FeN_4_, (Fe–Fe)­N_4_. Charge density differences between the substrate and adsorbed intermediates for (d) NiN_4_, (Ni–Ni)­N_4_, (e) CoN_4_, (Co–Co)­N_4_, and (f) FeN_4_, (Fe–Fe)­N_4_. The Bader values are annotated at fixed positions to indicate the net charge changes of different components: for the single-layer models (top row), the upper-right value denotes the net charge gained/lost by the adsorbate, Δ*q*_ads_, and the lower-left value denotes the net charge change on the metal center, Δ*q*_M_; for the bilayer MMC models (bottom row), the upper-right value likewise denotes Δ*q*_ads_, while the central and bottom values correspond to the net charge changes on the top-layer and bottom-layer metal sites, denoted as Δ*q*_M_^top^ and Δ*q*_M_^bottom^, respectively. Reaction free energy profile for (g) NiN_4_, (Ni–Ni)­N_4_, (h) CoN_4_, (Co–Co)­N_4_, (i) FeN_4_, (Fe–Fe)­N_4_ under constant potential. Yellow and cyan denote charge accumulation and depletion, respectively. The isosurface value is set to 0.003 e Å^–3^.

For the noncoupled NiN_4_ and (Ni–Ni)­N_4_ systems, their band structures and projected density of states (PDOS) are nearly identical. The Ni d orbitals exhibit negligible changes in orbital distribution, indicating that the coupling effect is minimal and the electronic configuration remains largely undisturbed. This observation explains the negligible difference in the η_ORR_ between NiN_4_ and (Ni–Ni)­N_4_.

In the weakly coupled CoN_4_ and (Co–Co)­N_4_ systems (Figure 4b), certain 3d orbitals of Co exhibit slight variations in their contributions to the PDOS, suggesting the presence of weak metal–metal interaction. However, this coupling does not significantly alter the band structure or electron occupancy, thus limiting its influence on catalytic performance.

In contrast, the moderately coupled (Fe–Fe)­N_4_ configuration (Figure 4c) exhibits pronounced orbital hybridization under the MMC model. Near the Fermi level, the band structure becomes more dispersive, and the PDOS reveals a more uniform distribution of Fe d states, indicating enhanced electronic delocalization and stronger orbital coupling. These electronic features improve charge transport and optimize intermediate adsorption, thereby significantly boosting ORR activity (increasing η_ORR_ from 0.54 to 0.95 V), which is in excellent agreement with experimental observations.

For the strongly coupled (Ru–Ru)­N_4_ system (Figure S17), a more substantial electronic reconstruction is observed. Compared with the mononuclear SL model RuN_4_, which exhibits sharp and localized states near the Fermi level, the MMC model presents more continuous bands with multiple band crossings, and the d-orbital PDOS becomes broader and smoother. These features reflect enhanced orbital hybridization and improved electron transport, which help mitigate excessive intermediate adsorption and reduce reaction barriers. Similar band and PDOS behaviors can be observed in other coupled systems (Figure S17), further validating the critical role of metal–metal coupling in governing the SAR in M–N–C catalysts.

More generally, the magnitude of MMC varies markedly among different transition metals, as evidenced by the distinct degrees of band and PDOS reconstruction across the surveyed SL and MMC models in Figure S17. This metal dependency can be rationalized by the intrinsic d-band structure and electron filling, which jointly determine the availability of near-Fermi d states and the energetic matching required for interlayer d–d hybridization. When the interacting d orbitals are sufficiently extended and partially occupied, stronger d–d coupling becomes accessible, leading to a more pronounced redistribution and broadening of d -derived states near the Fermi level; in contrast, metals with less favorable filling or more localized d states exhibit much weaker perturbations upon stacking. In the layered architecture, the neighboring N–C framework further provides a ligand-field and charge-regulation environment that can shift d-level alignment and orbital splitting, thereby modulating the effective d–d coupling strength. Consequently, MMC strength emerges as an intrinsic electronic-structure origin for the observed structure–activity variations in M–N–C catalysts within the MMC framework (Figure S13).

To further elucidate the charge-based origin of the ORR activity differences between the SL and the MMC, we compared the adsorption-induced charge density differences and the corresponding Bader charge analyses for *OH, *O and *OOH (Figure 4d–f). Overall, for the Ni and Co systems, introducing a bilayer counterpart leads primarily to localized charge redistribution within the metal–adsorbate bonding region, with only minor changes in the Bader-derived charge transfer, indicating that the bilayer architecture perturbs the Frontier electronic states and metal–oxygen interactions only weakly. In contrast, (Fe–Fe)­N_4_ under the MMC exhibits substantially stronger adsorption-induced charge reorganization together with a more pronounced charge response at the metal sites, consistent with an amplified polarization and charge-transfer process enabled by interlayer coupling. This charge-level evidence corroborates the coupling-induced reconstruction of Fe d states near the Fermi level revealed by the projected band structures (PBS)/PDOS, collectively indicating that metal–metal coupling reshapes the local electronic structure and thereby alters adsorption thermodynamics. Such effects provide a mechanistic basis for why the MMC substantially reduces the long-standing discrepancy between CHE-based predictions and experimentally observed activity in Fe–N–C catalysts.

To simulate realistically electrocatalytic reactions under experimental conditions, we employed the constant-potential method. Compared with the conventional constant-charge approach, the constant-potential method offers much improved physical consistency, enabling accurate characterization of energy barriers under varying electrochemical potentials. Accordingly, we performed constant-potential simulations for systems with different coupling strengths, including noncoupled (Ni–Ni), weakly coupled (Co–Co), moderately coupled (Fe–Fe), and strongly coupled (Ru–Ru) configurations, as illustrated in Figure 4g. Under *U* = 0.3 V, both (Ni–Ni)­N_4_ and NiN_4_ exhibit nearly identical limiting potentials for the O_2_ → OOH* step, indicating that the noncoupled configuration exerts minimal influence on the reaction pathway and electronic structure. For CoN_4_ and (Co–Co)­N_4_, we adopted the experimentally measured half-wave potential (*U* = 0.8 V) as the operating condition.^12^ As shown in Figure 4h, the O_2_ → OOH* step in (Co–Co)­N_4_ already reaches the limiting potential, confirming the accuracy of our constant-potential simulation. Even when coupling effects are considered, the computed results remain in strong agreement with experimental observations. For CoN_4_, the rate-determining step remains the desorption of *OH, with a barrier of only 0.11 eV, further demonstrating the consistency between the SL and the MMC under weak coupling. As depicted in Figure 4i, for the (Fe–Fe)­N_4_ system at a typical experimental potential of *U* = 0.9 V,^14^ the energy barrier is only 0.1 eV, much lower than that of FeN_4_ (0.34 eV). Although SL and MMC yield divergent results under this potential, the MMC prediction aligns more closely with experimental trends, indirectly validating its necessity in describing moderately coupled systems. Furthermore, in the strongly coupled RuN_4_ system (Figure S18), the energy barrier of (Ru–Ru)­N_4_ is only 0.17 eV at *U* = 0.7 V,^44^ significantly lower than the 0.84 eV observed for RuN_4_. This large discrepancy indicates that neglecting coupling effects leads to substantial errors in barrier prediction. These findings further underscore the superior accuracy and physical reliability of the MMC in predicting reaction barriers for electrocatalytic systems involving significant coupling effects.

### Experimental Validation of the Metal–Metal Coupling and Single-Layer Models

To translate directly the predicted MMC effect into an experimentally controllable analogue and to benchmark the model-selection criterion across different coupling regimes, a series of molecular catalysts were prepared by anchoring metal phthalocyanine (M = Fe, Co, Ni) onto atomically dispersed single metal sites embedded in a nitrogen-doped hollow carbon (NHCS) spheres matrix (Figure S19), including Fe-based catalysts (Fe_SA_-NHCS with isolated FeN_4_ moieties, FePc@Fe_SA_-NHCS with coupled (Fe–Fe)­N_4_ moieties), Co-based catalysts (Co_SA_-NHCS with isolated CoN_4_ moieties, CoPc@Co_SA_-NHCS with coupled (Co–Co)­N_4_ moieties), Ni-based catalysts (Ni_SA_-NHCS with isolated NiN_4_ moieties, NiPc@Ni_SA_-NHCS with coupled (Ni–Ni)­N_4_ moieties). Although this experimental platform is not intended to be a one-to-one structural equivalent of the idealized graphene-supported M-N_4_ model used in the theoretical calculations, it preserves the key local M-N_4_ coordination motif and provides a bilayer-like architecture for probing the effect of metal–metal coupling in an experimentally accessible manner. Specifically, M_SA_-NHCS was synthesized by using a facile double-solvent impregnation method and subsequent pyrolysis at 900 °C with melamine.^50,51^ The MPc@M_SA_-NHCS was obtained by introducing MPc molecules as the secondary active site into the M_SA_-NHCS matrix.^52^ As shown in Figure S20, X-ray diffraction (XRD) patterns show that no peak assignable to metal nanoparticles are observed for Fe_SA_-NHCS, Co_SA_-NHCS or Ni_SA_-NHCS. For MPc@M_SA_-NHCS, the peaks assignable to MPc molecular can be captured in XRD patterns, indicating that MPc is successfully introduced in M_SA_-NHCS. Transmission electron microscope (TEM) and high-resolution TEM (HRTEM) images also show that no obvious metal nanoparticles are observed in the six catalysts, indicating the atomic dispersion of Fe, Co and Ni species in Fe-based catalysts (Fe_SA_-NHCS and FePc@Fe_SA_-NHCS), Co-based catalysts (Co_SA_-NHCS and CoPc@Co_SA_-NHCS), and Ni-based catalysts (Ni_SA_-NHCS and NiPc@Ni_SA_-NHCS), respectively (Figures 5a,b and S21–S26). To investigate the atomic states of the M species in catalysts, aberration-corrected high-angle annular dark-field scanning transmission electron microscopy (AC HAADF-STEM) was conducted. As displayed in Figure 5c, numerous bright dots (marked with red circles) are attributed to Fe atoms and uniformly dispersed on the carbon matrix, since the Fe atoms is heavier than the C and N and therefore appear brighter in Z-contrast. The projected distance between two adjacent Fe atoms in Fe_SA_-NHCS is larger than 0.40 nm (Figure S21), indicating that the Fe species mainly exist as single atoms. As for FePc@Fe_SA_-NHCS (Figure 5c, d), a large proportion of Fe atoms are adjacent to each other and presented in the form of dual Fe atoms, with the Fe–Fe distance of ∼0.28 nm, which is well consistent with the coupled (Fe–Fe)­N_4_ models based on DFT.

**Figure 5:**
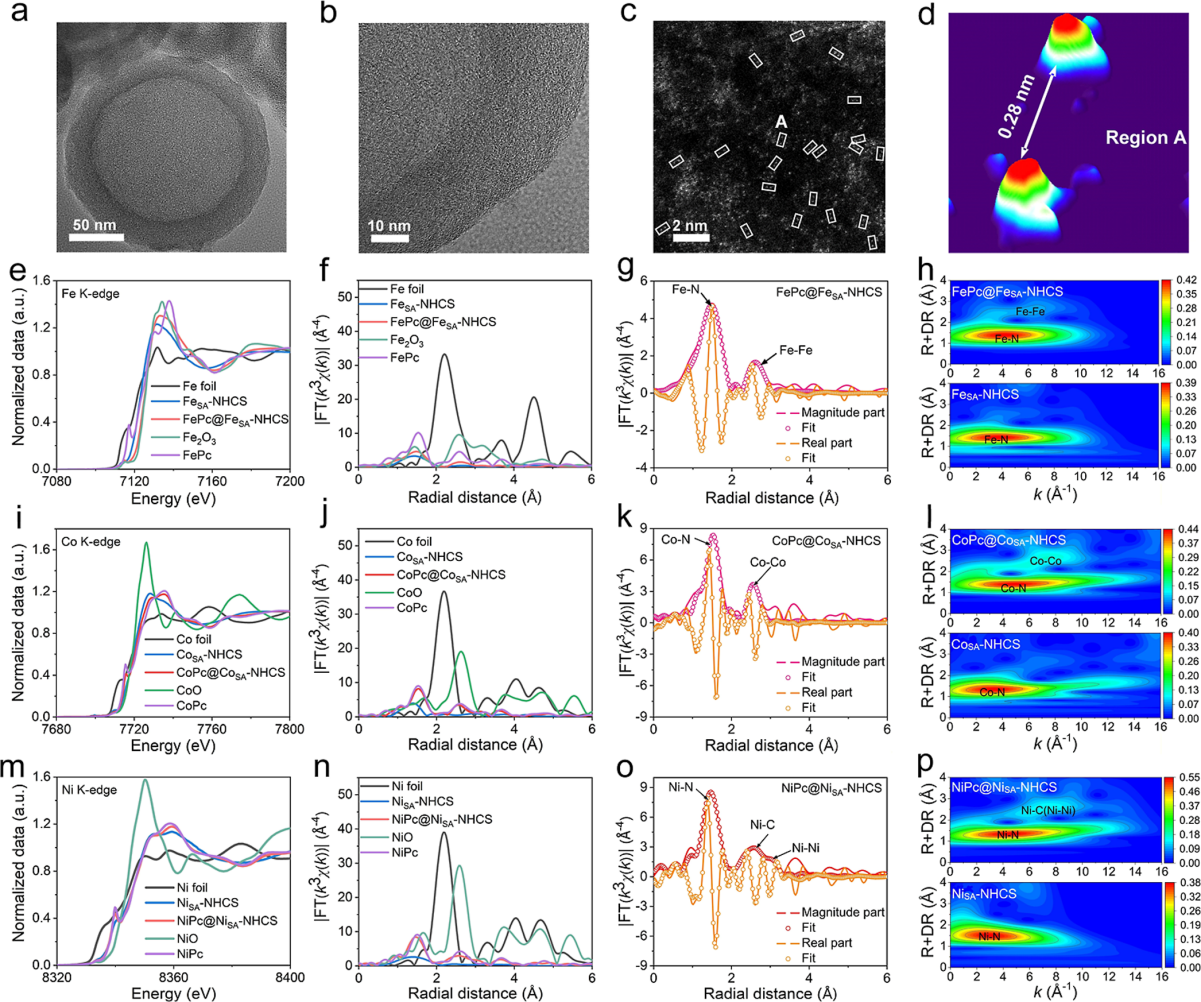
(a) TEM image, (b) HRTEM image, (c) magnified AC HAADF-STEM images for FePc@Fe_SA_-NHCS. The image clearly displays dual Fe atoms (white rectangle). (d) 3D atom-overlapping Gaussian-function fitting map of region A in (c) for the FePc@Fe_SA_-NHCS. (e) XANES and (f) *k* ^3^-weighted FT-EXAFS spectra of the FePc@Fe_SA_-NHCS and Fe_SA_-NHCS at the Fe *K*-edge, accompanied by the Fe foil, Fe_2_O_3_, FePc as the reference samples. (g) The corresponding *k* ^3^-weighted EXAFS fitting curves at R space for FePc@Fe_SA_-NHCS. (h) WT-EXAFS of FePc@Fe_SA_-NHCS and Fe_SA_-NHCS. (i) XANES and (j) *k* ^3^-weighted FT-EXAFS spectra of the CoPc@Co_SA_-NHCS and Co_SA_-NHCS at the Co *K*-edge. (k) The corresponding *k* ^3^-weighted EXAFS fitting curves at R-space for CoPc@Co_SA_-NHCS. (l) WT-EXAFS of CoPc@Co_SA_-NHCS and Co_SA_-NHCS. (m) XANES and (n) *k* ^3^-weighted FT-EXAFS spectra of the NiPc@Ni_SA_-NHCS and Ni_SA_-NHCS at the Ni *K*-edge. (o) The corresponding *k* ^3^-weighted EXAFS fitting curves at R-space for NiPc@Ni_SA_-NHCS. (p) WT-EXAFS of NiPc@Ni_SA_-NHCS and Ni_SA_-NHCS.

X-ray absorption spectroscopy (XAS) was employed to further analyze the detailed local atomic microstructures of the six catalysts. As for Fe-based catalysts (Fe_SA_-NHCS and FePc@Fe_SA_-NHCS), the X-ray absorption near edge structure (XANES) spectra in Figure 5e show that the XANES curves for Fe_SA_-NHCS and FePc@Fe_SA_-NHCS lie between those for Fe foil and Fe_2_O_3_, indicating that their Fe atoms exhibit a positive valence. Fourier transform (FT) *k* ^3^-weighted extended X-ray absorption fine structure (FT-EXAFS) spectra at R-space show a main peak at 1.45 Å that corresponds to the Fe–N shell (relative to FePc) is captured for Fe_SA_-NHCS and FePc@Fe_SA_-NHCS (Figure 5f). To determine the detailed coordination structure of the Fe atoms, theoretical calculations and fitted EXAFS at the Fe K-edge were conducted. As shown in Figures 5g, S27 and Table S2, both Fe_SA_-NHCS and FePc@Fe_SA_-NHCS afford an average Fe–N coordination number of 3.8, indicating the presence of FeN_4_ structure in both catalysts. In addition, the FePc@Fe_SA_-NHCS provides an Fe–Fe coordination number of 1.0 with the average bond length of 2.8 Å, meaning that most FeN_4_ structure in Fe_SA_-NHCS are preferentially bonded with FePc to form the coupled (Fe–Fe)­N_4_ models (inset of Figure 5g). To further verify the structure in FePc@Fe_SA_-NHCS, DFT calculations were first employed to investigate the possible structures (7 models) containing the in plane dual sites models, Fe–Fe MMC models and laterally shifted bilayer registries of Fe–Fe MMC models. First, the formation energy of series of possible structures were calculated (Figure S28), in which the model 1 (Fe–Fe MMC) has the lowest formation energy of −5.63 eV, indicating that the model 1 was the most stable structure. Moreover, we have added a comparison between the simulated XANES spectra of the possible structures and the experimental spectra. It is noted that the simulated spectra based on the Fe–Fe MMC structure (model 1) agree well with the experimental XANES results of FePc@Fe_SA_-NHCS, indicating that this model 1 is the most likely structure in FePc@Fe_SA_-NHCS (Figure S29 and Table S3). Figure 5i–k, m-o displays that the Co atoms in Co_SA_-NHCS exist in a form of CoN_4_ structure and the Ni atom in Ni_SA_-NHCS exist in a form of NiN_4_ structure. After the introduction of MPc molecular, the CoPc@Co_SA_-NHCS is mainly composed of coupled (Co–Co)­N_4_ moieties with the average bond length of 2.9 Å and the NiPc@Ni_SA_-NHCS is mainly composed of coupled (Ni–Ni)­N_4_ moieties with the average bond length of 3.5 Å (Figure 5k,o). Further support for this conclusion can be provided by the simulated EXAFS spectra based on the coupled (M–M)­N_4_ moieties, which are consistent with the experiment spectra (Figures S30 and S31), and the corresponding wavelet transform (WT) contour plots (Figures 5h,l,p and S32–S34). These results further demonstrate the successful synthesis of coupled (M–M)­N_4_ moieties in MPc@M_SA_-NHCS.

Subsequently, to experimentally validate the theory-predicted activity trend and, more importantly, the coupling-strength dependence of MMC, the ORR activities of the six catalysts were evaluated in alkaline media using a rotating ring-disk electrode (RRDE). The LSV curves in Figure 6a reveal a clear metal-selective response upon introducing the bilayer-like coupled motif. For Fe, constructing the coupled (Fe–Fe)­N_4_ environment in FePc@Fe_SA_-NHCS induces a pronounced positive shift in both the onset potential and half-wave potential (*E*_onset_ = 0.98 V; *E*_1/2_ = 0.88 V) relative to the isolated-site analogue Fe_SA_-NHCS (*E*_onset_ = 0.92 V; *E*_1/2_ = 0.78 V). Moreover, the smaller Tafel slope of FePc@Fe_SA_-NHCS (30.7 mV dec^–1^) than that of Fe_SA_-NHCS (40.3 mV dec^–1^) indicates optimized catalytic activity and ORR kinetics for the former. These results demonstrate that Fe–Fe coupling is an important active site configuration for ORR energetics. In sharp contrast, Co- and Ni-based catalysts show essentially unchanged activity after coupling: CoPc@Co_SA_-NHCS exhibits the same *E*_onset_ and *E*_1/2_ as Co_SA_-NHCS (*E*_onset_ = 0.89 V; *E*_1/2_ = 0.77 V for both) accompanied by the same Tafel slope (∼36.5 mV dec^–1^) and NiPc@Ni_SA_-NHCS is likewise indistinguishable from Ni_SA_-NHCS (*E*_onset_ = 0.82 V; *E*_1/2_ = 0.69 V; Tafel slope of ∼74.9 mV dec^–1^ for both, Figure 6b,c). This side-by-side comparison disentangles the role of the “second metal center” and indicates that, in weak/noncoupled regimes, the introduced neighboring site does not measurably perturb the operative adsorption thermodynamics.

**Figure 6:**
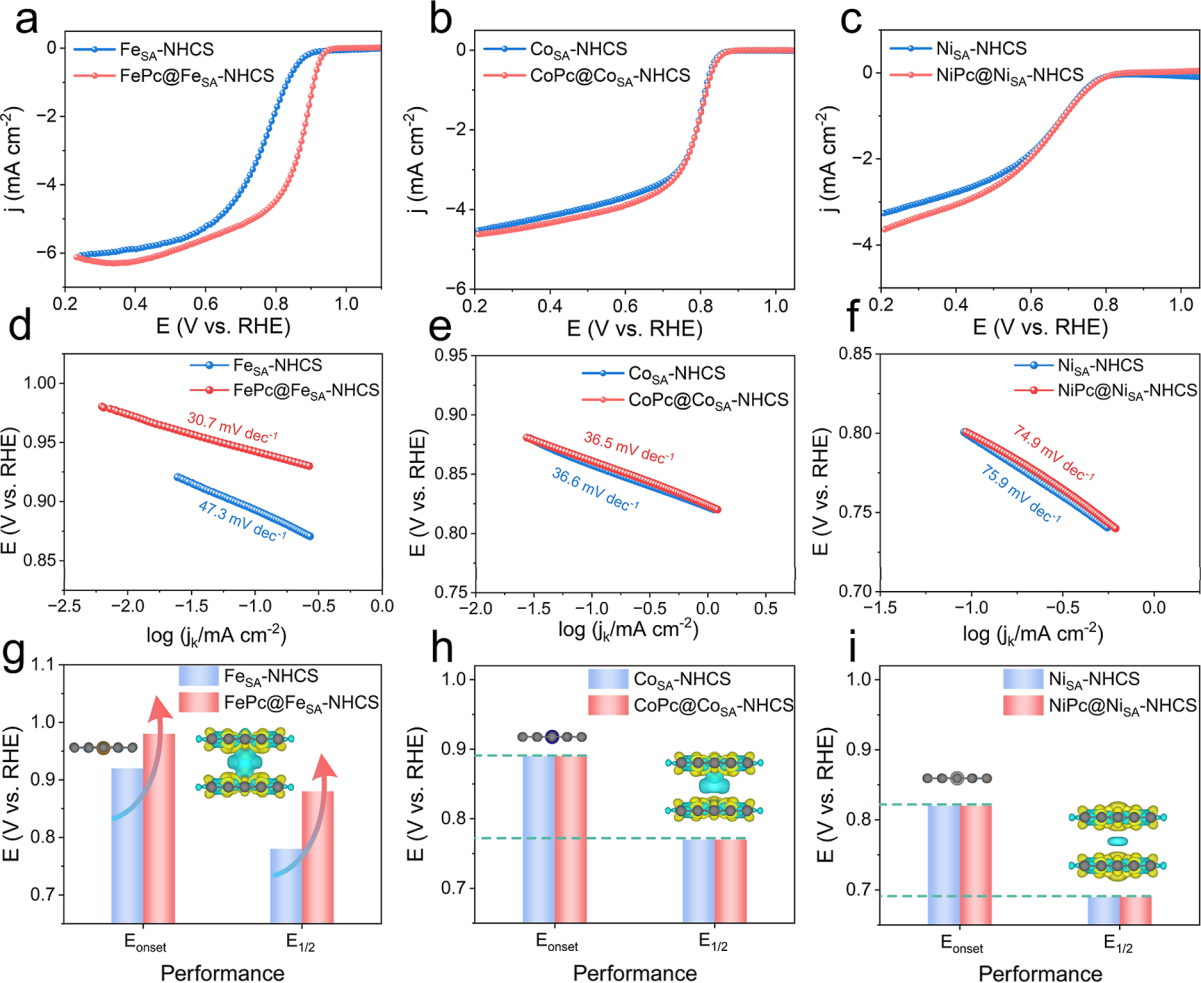
ORR polarization curves in 0.1 M KOH for (a) Fe-based catalysts, (b) Co-based catalysts and (c) Ni-based catalysts. The corresponding Tafel plots and comparisons for (d,g) Fe-based catalysts, (e,h) Co-based catalysts and (f,i) Ni-based catalysts.

Collectively, these cross-metal control experiments establish a direct correspondence between coupling strength and model fidelity: for the moderately coupled Fe system (Figure 6g), the experimentally observed *E*_1/2_ is in close agreement with the MMC-predicted limiting potential, whereas the conventional SL description would substantially underestimate the activity, confirming that coupling must be explicitly included for accurate prediction. Conversely, for Co and Ni (Figure 6h,i), the experimentally identical activities between isolated and coupled motifs mirror the minimal MMC-SL difference predicted under weak coupling, which also rationalizes why CHE-based SL calculations already exhibit good agreement with experiments for CoN_4_ and NiN_4_. Overall, the RRDE results provide a self-consistent validation of the MMC framework and the coupling-guided model-selection criterion, laying a quantitative experimental basis for subsequent descriptor-driven screening and rational construction of high-activity coupled motifs.

## Conclusions

This study proposes a possible mechanistic explanation for the discrepancy between theoretical predictions and experimental observations of ORR in single-atom TM–N–C catalysts, identifying an interlayer metal–metal coupling configuration as an important structural case that can influence the multielectron transfer ORR process. MMC strength is shown to be an important descriptor to determine if the conventional SL model or MMC prevails.

Guided by XAFS evidence indicating an additional coordination feature beyond the canonical M–N shell in Fe–N–C, we show that stacked bilayer MMC can help reconcile the long-standing mismatch between theory and experiment for Fe–N–C and reveal that coupling-induced electronic reconstruction, interfacial polarization, and local field focusing play important roles in reshaping the adsorption thermodynamics and reaction barriers.

By systematically benchmarking a broad set of transition-metal systems, we further establish a coupling-strength-guided model-selection criterion that delineates when MMC is required versus when SL remains sufficient. Importantly, this coupling-aware perspective naturally defines a mixed SL + MMC activity landscape that preserves continuous scaling relations and volcano-type trends, enabling meaningful cross-system comparison. Building on this foundation, we extend the framework with machine learning and data mining to identify local geometric and electronic descriptors that govern activity within the mixed space, thereby enabling quantitative SARs that are transferable and practically useful for catalyst screening and rational design.

Crucially, the theoretical picture is validated experimentally using a bilayer-like catalyst platform that enables controlled construction of isolated MN_4_ motifs and coupled (M–M)­N_4_ environments. Multitechnique structural characterization, including EXAFS-based fitting, supports the formation of the intended coupled motifs, while electrochemical measurements demonstrate a metal-dependent response that mirrors the coupling criterion: Fe exhibits a pronounced enhancement upon coupling, whereas Co and Ni remain essentially unchanged, consistent with their weak or negligible coupling regimes. Together, these results establish a self-consistent link between real-space structure, local electronic reconstruction, and macroscopic ORR performance. The MMC, coupled with the coupling-guided modeling protocol and ML-derived descriptors, offers a unified and predictive strategy for understanding, benchmarking, and designing next-generation TM–N–C ORR catalysts. This strategy may be further extended to heteronuclear MMC systems, where mixed-metal coupling could provide an additional and tunable design space for modulating coupling strength and catalytic activity.

## Supplementary Material



## Notes

The Supporting Information contains additional computational and experimental details, including DFT methods, computational hydrogen electrode and constant-potential frameworks, DFT + U, solvation, and AIMD calculations, dissolution-potential and formation-energy calculations, subgroup discovery and machine-learning protocols, catalyst synthesis, characterization methods, electrochemical measurements, supplementary figures, calculated and experimental ORR activity comparisons, and EXAFS fitting parameters (PDF)
