# Supplementary material for: Strength of Interlayer Metal–Metal Coupling as Key Active Site Configuration and Atomic Descriptor for Single-Atom Catalysts
Source: J Am Chem Soc. 2026 Jul 9;148(28):29895–907. doi: 10.1021/jacs.6c04989 (PMC13397547; doi:10.1021/jacs.6c04989)
Supplement: Supplementary file 1 [file ja6c04989_si_001.pdf]

Strength of Interlayer Metal-Metal Coupling as Key Active Site Configuration and Atomic Descriptor for Single-Atom Catalysts

Liangliang Xu[1,4,+,*], Jiankang Wang[1,+], Hanxu Yao[2], Jinpei Huang[3], Zijing Li[1], Xingkun Wang[2,*], Linguo Lu[4,*], Jian Zhou[5], Peixin Cui[6], Heqing Jiang[2], Zhengxiao Guo[1,7,*]

[1]Department of Chemistry, The University of Hong Kong, Hong Kong SAR, 999077, China

[2]State Key Laboratory of Photoelectric Conversion and Utilization of Solar Energy, Qingdao New Energy Shandong Laboratory, Qingdao Institute of Bioenergy and Bioprocess Technology, Chinese Academy of Sciences, Qingdao, 266100, P. R. China

[3]Graduate School of Advanced Imaging Science, Chung-Ang University, Seoul, 06974, South Korea

[4]Department of Chemistry, University of Puerto Rico, Rio Piedras, San Juan, PR 00931, United States

[5]Physical Chemistry, University of Konstanz, Universitätsstraße 10, Box 714, Konstanz 78457, Germany

[6]State Key Laboratory of Soil & Sustainable Agriculture, Institute of Soil Science, Chinese Academy of Sciences

[7]Hong Kong Quantum AI Lab, AIR@InnoHK of Hong Kong Government, Hong Kong SAR, 999077, China

[+]These authors contributed equally to this work.

*Corresponding author: xuliang@hku.hk; wangxk@qibebt.ac.cn; linguo.lu@upr.edu; zxguo@hku.hk

Method

Density functional theory (DFT)

Computational hydrogen electrode (CHE) framework

The spin-polarized DFT calculations were performed using the plane-wave projector augmented-wave (PAW) method, applying the semi-local Perdew-Burke-Ernzerhof (PBE) exchange-correlation functional, as implemented in the Vienna *ab* initio simulation package (VASP).[1-3] A plane-wave cutoff energy of 450 eV was used. The Brillouin zone is sampled with 2×2×1 and 4×4×1 *k*-point mesh for geometry optimization and electronic property calculations, respectively. The structures were first relaxed using conjugate-gradient algorithm, until energy and forces on each atom were converged below 10^{-5} eV/atom and 0.02 eV/Å, respectively. In addition, the van der Waals (vdW) interactions were described by the Grimme's DFT-D3 scheme.[4] Additional calculations were performed to evaluate the influence of DFT+U correction and solvation effects on the ORR free-energy profiles. The DFT+U correction was applied using the rotationally invariant Dudarev approach, where U_{eff}=U−J. The Hubbard correction was applied only to the 3*d* orbitals of Fe, Co, and Ni atoms. The U_{eff} values were set to 4.04, 3.52, and 3.40 eV for Fe, Co, and Ni, respectively, following previous reports for Co,

Fe, and Ni systems.[5-7] Implicit solvation effects were considered using VASPsol, in which the aqueous environment was described by a continuum solvation model. The same ORR intermediates and reaction steps were recalculated under DFT+U and implicit solvation conditions. The resulting free-energy profiles were compared with those obtained from the original calculations to assess the sensitivity of the limiting potential and potential-limiting step to these methodological factors. To further evaluate the thermal stability of the optimized catalyst structures, ab initio molecular dynamics (AIMD) simulations were carried out using VASP. The AIMD simulations were performed in the canonical ensemble using the Nosé-Hoover thermostat at 300 K. A time step of 1 fs was used, and the total simulation length was 10 ps.

To evaluate the electrochemical stability of the metal centers in the TMN_4-SL and (TM-TM)N_4-MMC models, the dissolution potential was calculated as follows:

$$U_{\mathrm{diss}} = U_{\mathrm{diss}}^{0}(\mathrm{M}) - \frac{E_f}{ze}$$

where $U_{\mathrm{diss}}^{0}(\mathrm{M})$ is the standard dissolution potential of the corresponding bulk metal M, z is the number of electrons involved in the dissolution process, and e is the elementary charge. For the single-layer TMN4-SL model, the formation energy of the metal center was calculated as:

$$E_f = E_{\mathrm{TMN_4\text{-}SL/graphene}} - E_{\mathrm{N_4\text{-}SL/graphene}} - \frac{E_{\mathrm{bulk}}(\mathrm{M})}{N_{\mathrm{bulk}}}$$

where $E_{\mathrm{TMN_4\text{-}SL/graphene}}$ is the total energy of the TMN_4-SL model, $E_{\mathrm{N_4\text{-}SL/graphene}}$ is the total energy of the corresponding N_4-doped graphene framework after removing the metal atom, $E_{\mathrm{bulk}}(\mathrm{M})$ is the total energy of the bulk metal supercell, and N_{bulk} is the number of metal atoms in the bulk metal supercell. Thus, $E_{\mathrm{bulk}}(\mathrm{M})/N_{\mathrm{bulk}}$ represents the energy per atom of bulk metal M.

For the MMC model, the average formation energy per metal atom was calculated as:

$$E_f = E_{\mathrm{(TM\text{-}TM)N_4\text{-}MMC/graphene}} - E_{\mathrm{N_4\text{-}MMC/graphene}} - N_{\mathrm{M}} \frac{E_{\mathrm{bulk}}(\mathrm{M})}{N_{\mathrm{bulk}}}$$ where

$E_{\mathrm{(TM\text{-}TM)N_4\text{-}MMC/graphene}}$ is the total energy of the MMC, $E_{\mathrm{N_4\text{-}MMC/graphene}}$ is the total energy of the corresponding metal-free bilayer N-doped graphene framework, and N_{M} is the number of metal atoms removed from the MMC. For the homonuclear (TM-TM)N_4-MMC

considered in this work, $N_M = 2$.

The Gibbs free energy of adsorption was calculated using the following equation:

$$\Delta G = E_{DFT} + ZPE - TS$$

where ΔG represents Gibbs free energy, the E_{DFT} is the DFT adsorption energy, ZPE and TS are vibrational zero-point energy and entropy contributions, respectively.

The ORR mechanism considered is as follows:

$$O_2 + H_2O + e^- \rightarrow OOH^* + OH^-$$

$$OOH^* + e^- \rightarrow O^* + OH^-$$

$$O^* + H_2O + e^- \rightarrow OH^* + OH^-$$

$$OH^* + e^- \rightarrow * + OH^-$$

The Gibbs free energy changes (ΔG) for each elementary step were calculated using the following equations:

$$\Delta G_1 = 4.92 - \Delta G_{OOH*}$$

$$\Delta G_2 = \Delta G_{OOH*} - \Delta G_{O*}$$

$$\Delta G_3 = \Delta G_{O*} - \Delta G_{OH*}$$

$$\Delta G_4 = \Delta G_{OH*}$$

where OOH*, O*, and OH* are the ORR intermediates adsorbed on the catalyst surface.

The ORR limiting potential was calculated as:

$$\eta_{ORR} = -\frac{MAX(\Delta G_1, \Delta G_2, \Delta G_3, \Delta G_4)}{e} \text{ V}$$

Constant potential framework

The reaction energetics were evaluated with a grand-canonical DFT framework as developed by Xia et al.[8] For each reaction intermediate, the electron number was iteratively adjusted to converge the electronic chemical potential corresponding to the target electrode potential U. The adsorption free energy at a fixed potential was then obtained from the grand free energy change:

$$\Delta\Omega = \Delta G - \mu_e \Delta N$$

where ΔN is the net charge change relative to the corresponding potential, and the electron chemical potential μ_e is related to the applied potential by:[9]

$$\mu = \mu_{SHE} - eU_{SHE}$$

where μ_{SHE} denotes the chemical potential of the standard hydrogen electrode (SHE), which is set to -4.44 eV, in agreement with the experimental value relative to vacuum (-4.44 ± 0.02 eV).[10] U_{SHE} is the electrode potential compared to the SHE.

The Landau free energy changes for each elementary step were calculated by[11]

$$\Delta\Omega_1 = 4.92 - \Delta\Omega_{\mathrm{OOH}*} + 4eU$$

$$\Delta\Omega_2 = \Delta\Omega_{\mathrm{OOH}*} - \Delta\Omega_{\mathrm{O}*} + 3eU$$

$$\Delta\Omega_3 = \Delta\Omega_{\mathrm{O}*} - \Delta\Omega_{\mathrm{OH}*} + 2eU$$

$$\Delta\Omega_4 = \Delta\Omega_{\mathrm{OH}*} + eU$$

Data Mining

Subgroup discovery (SGD)

The subgroup discovery was performed using RealKD package.[12] Each feature was split to 14 subsets using 14-means clustering algorithm.[13] The borders between adjacent data clusters (a1, a2, ...) are applied further for construction of inequalities (feature1 < a1), (feature2 ≥ a2), etc. While final result might depend on the number of considered clusters, in our previous study we found that relatively high numbers of considered clusters provide essentially the same result.[14] The candidate subgroups are built as conjunctions of obtained simple inequalities. The main idea of SGD is that the subgroups are unique if the distribution of the data in them is as different as possible from the data distribution in the whole sampling. Here the data distribution is the distribution of a target property (η_{ORR}). The uniqueness is evaluated with a quality function.[15] In this study we used the following function:

$$Q = \frac{N_{sub}}{N_{whole}} \frac{med_{whole} - med_{sub}}{med_{whole} - min_{whole}} (1 - \frac{amd_{sub}}{amd_{whole}})$$

where N_{sub} and N_{whole} are the numbers of materials in the subgroup and the whole data set, respectively, med_{sub}, med_{whole} denote median value of target properties in the subgroup and whole data set, respectively, min_{whole} is the minimum value of target properties in the whole data set, amd_{sub}, amd_{whole} denote absolute average deviation of the data around the median of target properties in the subgroup and whole data set, respectively. With this function the algorithm is searching for subgroups with lower values of target properties. The search was done with an adapted for such purposes Monte-Carlo algorithm,[16] in which first a certain

number of trial conjunctions (seeds) is generated. Afterwards, for each seed (accompanied with pruning of inequalities) the quality function is calculated. We have tested here several numbers of initial seeds: 640 000, 6 400 000, 64 000 000, and 640 000 000. The subgroups with the overall high quality function value were selected.

Machine learning

The complete dataset was evaluated using a repeated random resampling protocol: in each iteration, three samples (approximately 20% of the dataset) were randomly held out as an independent test set and the remaining samples were used for training; this procedure was repeated 20 times with different random seeds. For each iteration, a Gradient Boosting Regression (GBR) model was trained, and its key hyperparameters (e.g., learning rate, maximum tree depth and subsampling-related parameters) were optimized by randomized search with 3-fold cross-validation on the training set (500 sampled hyperparameter combinations). Model performance was assessed on the held-out test set using the root mean square error (RMSE). To quantify feature importance, SHAP (Shapley Additive Explanations) was applied post hoc to the optimized model from each iteration; mean absolute SHAP values were computed for all descriptors and used to rank their contributions, and the resulting rankings were aggregated over the 20 resampling runs to assess stability. All analyses were implemented in Python using scikit-learn, SHAP, pandas, NumPy, and matplotlib.

Experiments

Preparation of hollow polymer spheres (HPS). The HPS precursor was synthesized using the following method. Specially, solution A containing 0.375 mM Pluronic P123 and 12 mM SO was injected into solution B containing 8.3 mM HMT and 20 mM DA. The mixed solution was transferred to a Teflon-lined stainless-steel autoclave and heated to 160 ℃ for 2 h. Finally, the HPS precursors were obtained by centrifuging with water and dried at 50 °C for 24 h.

Synthesis of M_{SA}-NHCS with MN_4 atomic structure (M=Fe, Co, Ni). HPS (50 mg) was dispersed in n-pentane (10 mL) by ultrasonication for 30 min and stirred for 30 min at room temperature. A certain amount of M^{n+} solution ($FeCl_3 \cdot 6H_2O$, $CoCl_2 \cdot 6H_2O$, $NiCl_2 \cdot 6H_2O$) was injected into the HPS solution and then continuously stirred for 8 h at room temperature

to evaporate the solvent. The precursor was obtained after drying in a vacuum at 50 ℃ for 24 h. The obtained precursor and melamine with the ratio of 1:10 were ground together and heated at 900 ℃ for 30 min under Ar atmosphere to yield the M_{SA}-NHCS catalysts.

Synthesis of MPc@M_{SA}-NHCS with coupled (M-M)N_4 atomic structure (M=Fe, Co, Ni). 50 mg of M_{SA}-NHCS was uniformly dispersed in N,N-Dimethylformamide (DMF) and ethanol solution (4:1), and then a certain amount of MPc was dispersed in DMF and ethanol solution (1:1). The MPc solution was injected into M_{SA}-NHCS solution and kept under magnetic stirring for 24 h. Then the mixed solution was centrifuged by ethanol and dried under vacuum overnight to obtain the precursor. Then, the obtained precursor was annealed at 350 ℃ under Ar atmosphere for 2 h to yield the MPc@M_{SA}-NHCS catalysts.

Characterization. The X-ray diffraction (XRD) patterns were recorded using a Bruker D8 Advance diffractometer. High-resolution TEM (HRTEM), high-angle annular dark-field scanning transmission electron microscopy (HAADF-STEM), and energy-dispersive X-ray spectroscopy (EDS) were performed using a FEI Talos F200S microscope. Aberration-corrected (AC) HAADF-STEM was employed on a Titan-Cubed Themis G2. The X-ray absorption fine structure spectra (Co, Ni and Fe K-edge) were collected at beamline BL14W1 and the data collection was carried out in fluorescence excitation mode using a Lytle detector for samples. All spectra were collected in ambient conditions.

Electrochemical measurements. The ORR performances were investigated using an electrochemical station (CHI-842) equipped with a conventional three-electrode system. A catalyst-modified glassy carbon electrode (GCE), a Pt wire, and an Ag/AgCl (KCl-saturated) electrode were used as the working, counter, and reference electrodes, respectively. 2 mg catalysts were added to 1 mL Nafion-solution and ultrasonicated for 1 h. Then, 42 μL of the suspension was pipetted onto a polished rotating ring disk electrode (RRDE, diameter: 5 mm), which was used as the working electrode. Linear sweep voltammetry (LSV) was used to explore the ORR performance in O_2-saturated 0.1 M KOH at a rotation rate of 1600 rpm.

Supplemental Figures and Tables

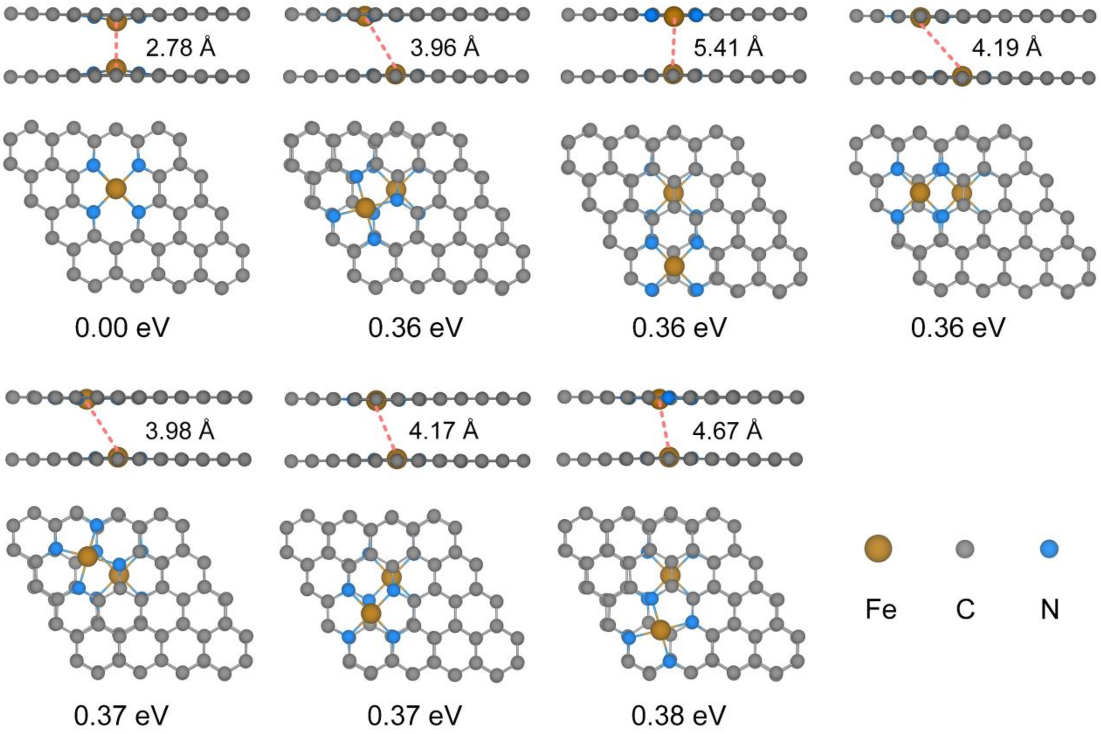

Figure S1. Relative energies of MMC configurations with different stacking types.

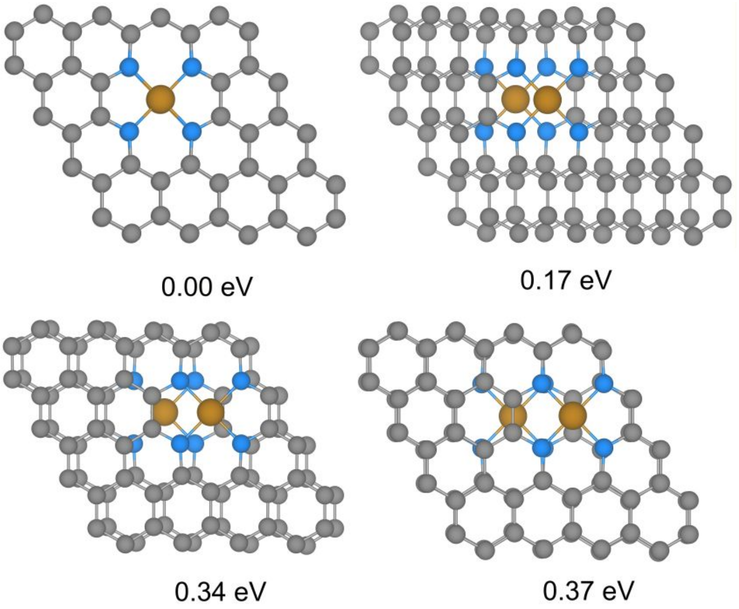

Figure S2. Representative laterally shifted bilayer registries considered for the Fe-Fe MMC model and their relative energies after structural optimization.

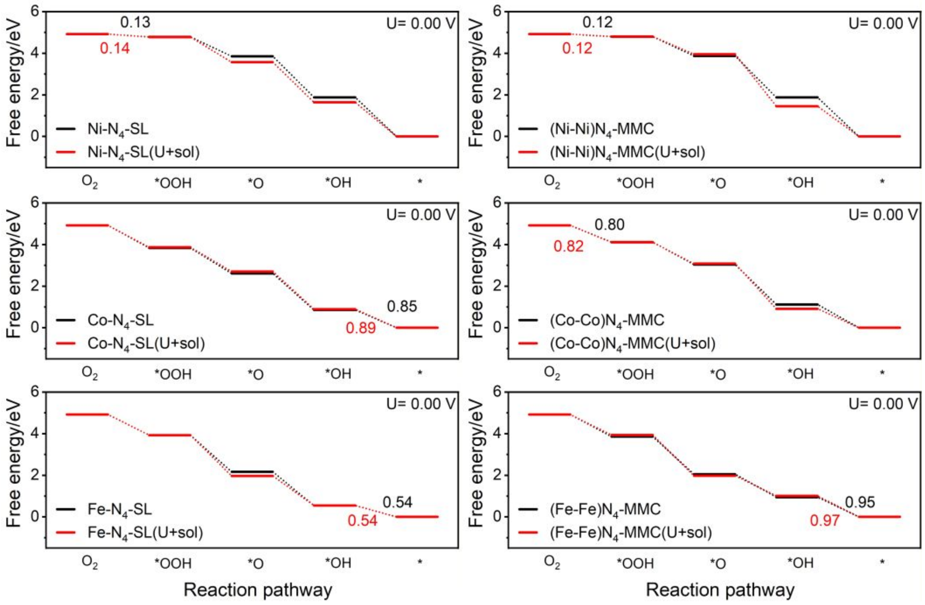

Figure S3. Comparison of ORR free-energy diagrams for Fe-, Co-, and Ni-based MMC and SL models with and without DFT+U correction and solvation effect.

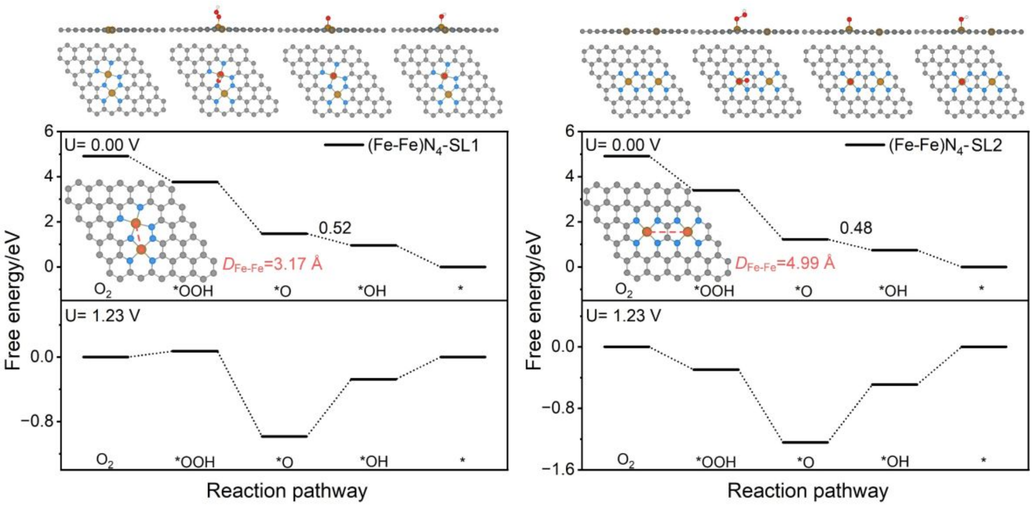

Figure S4. Models and free energy diagrams of single-layer (Fe-Fe)N_4-SL configurations with varying Fe-Fe distances.

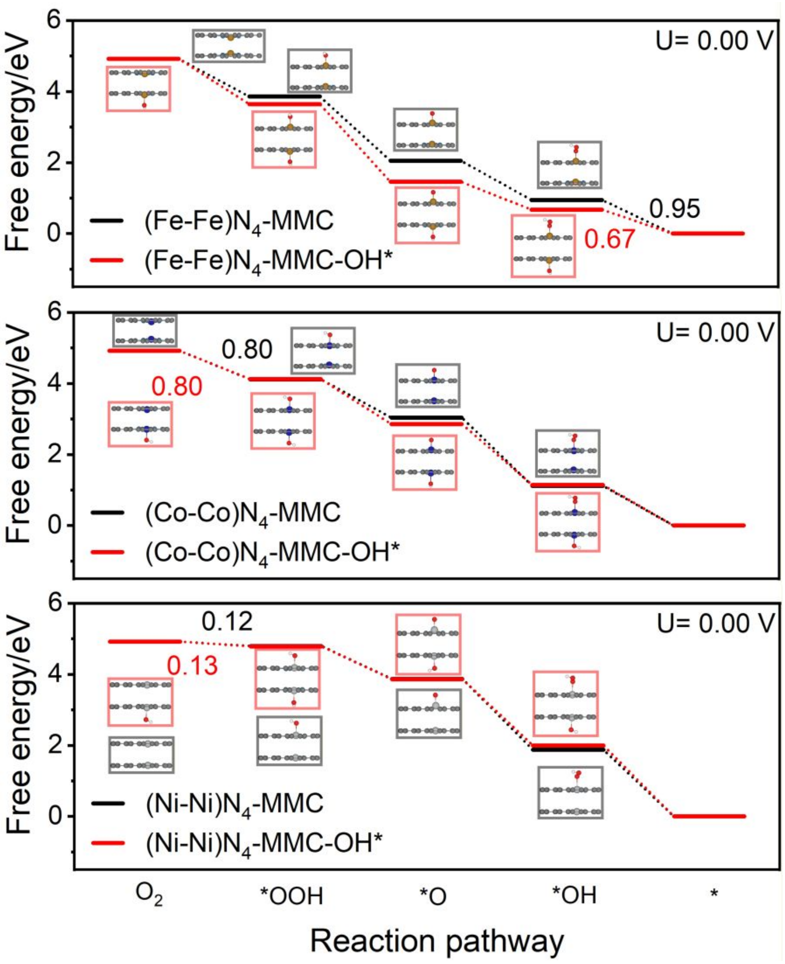

Figure S5. Comparison of ORR free-energy diagrams for bare and OH-ligated MMC models of (Fe-Fe)N_4, (Co-Co)N_4, and (Ni-Ni)N_4.

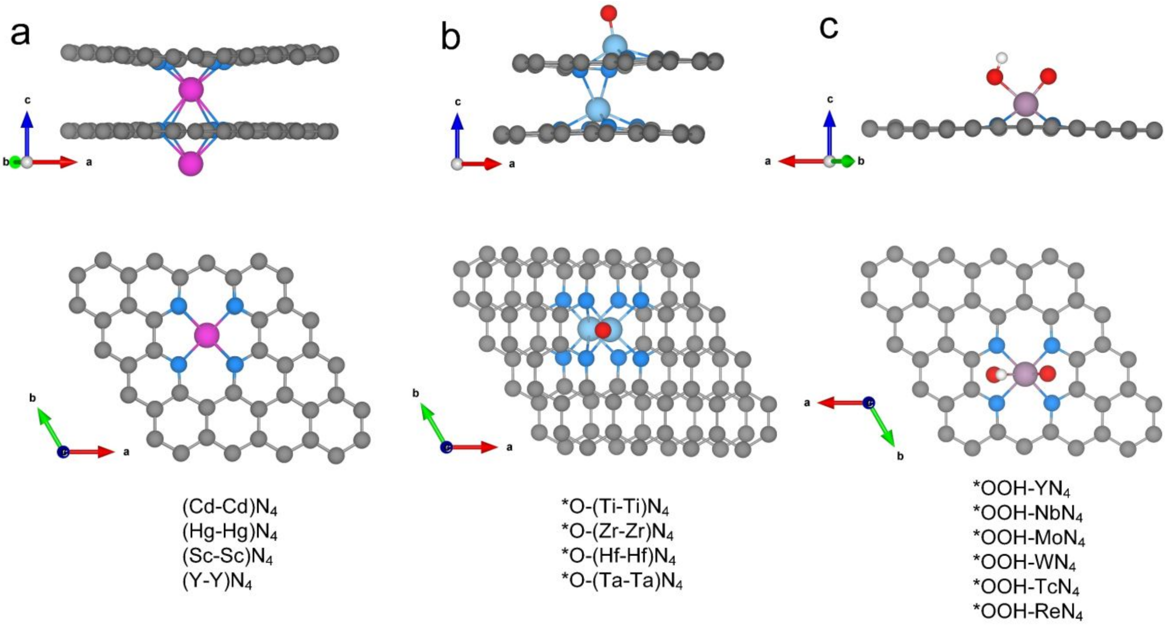

Figure S6. (a) MMC with the formation of M-N_8 coordination. (b) MMC exhibiting stacking fault

formation after O adsorption. (c) SL showing spontaneous OOH dissociation upon adsorption.

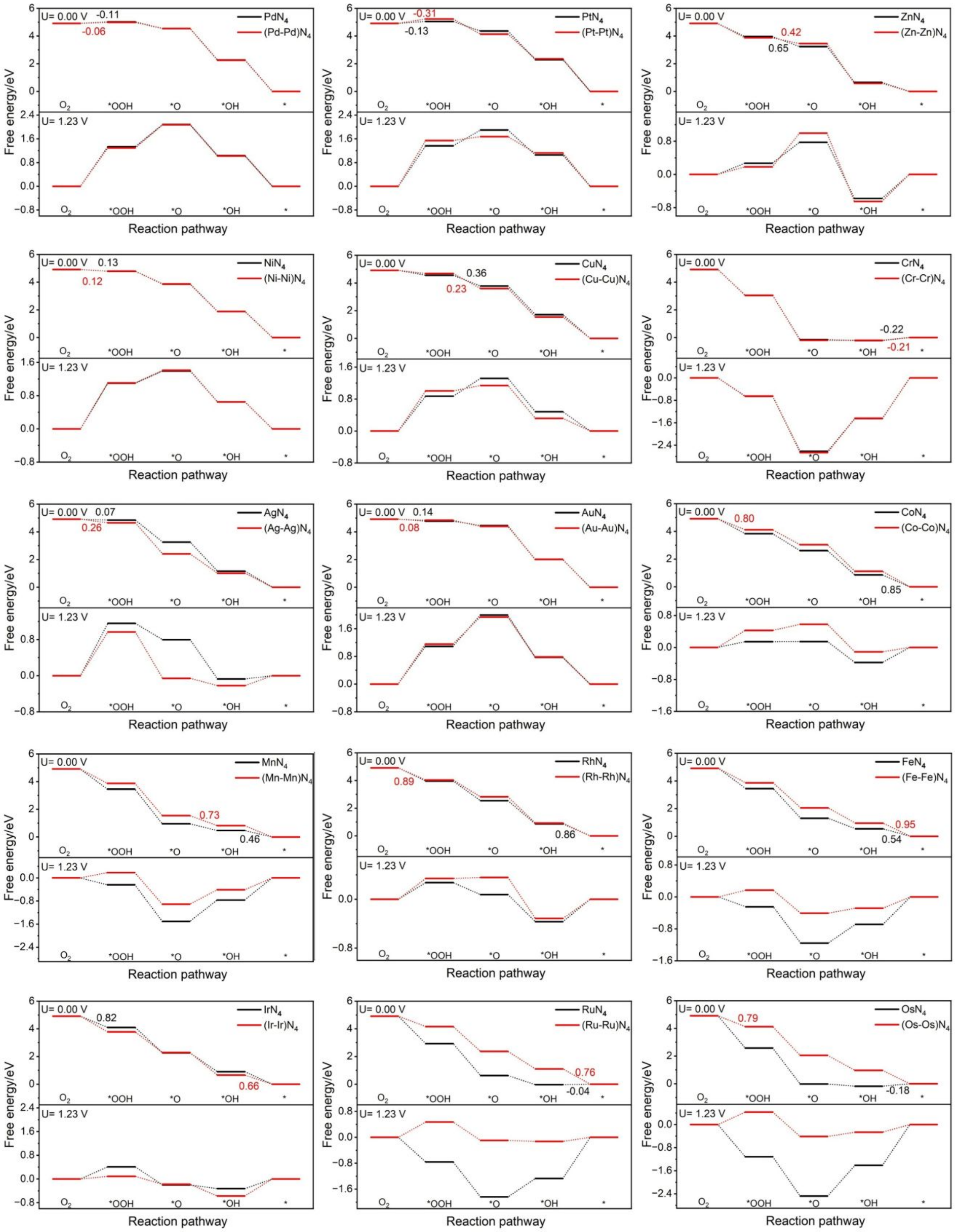

Figure S7. Free energy diagram of MN_4-SL and $(M\text{-}M)N_4$-MMC configurations.

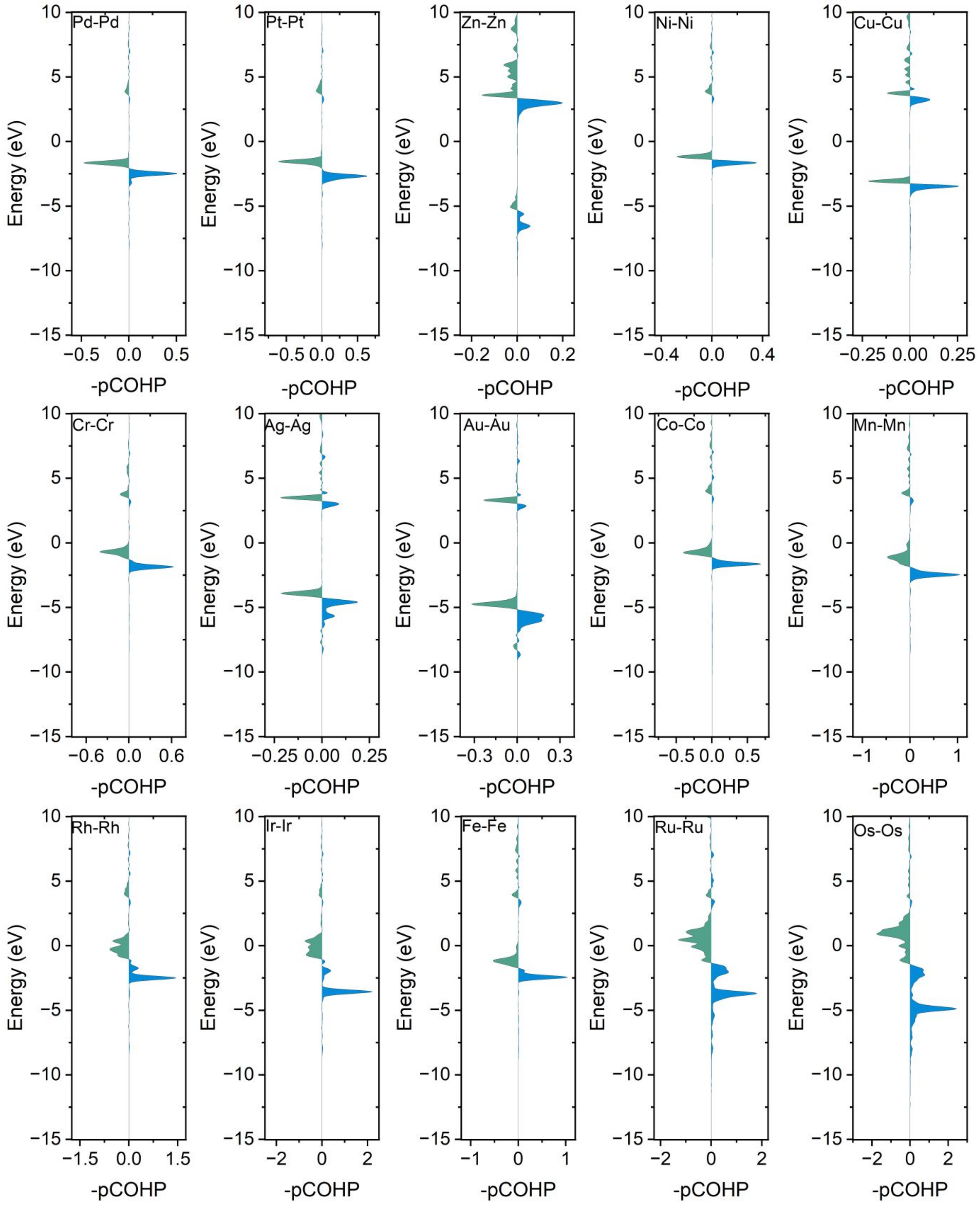

Figure S8. Crystal orbital Hamilton population (COHP) of MMC configurations.

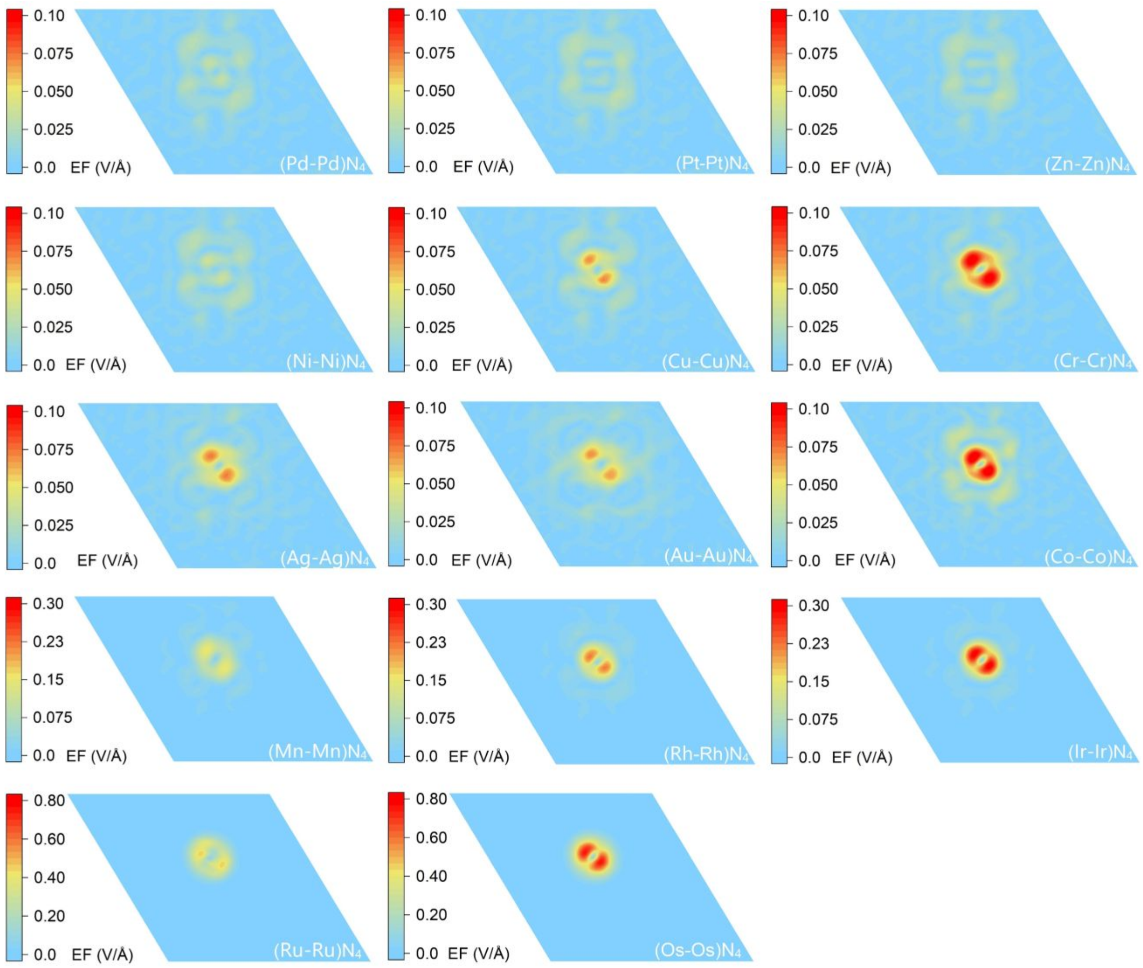

Figure S9. Electric field of MMC configurations.

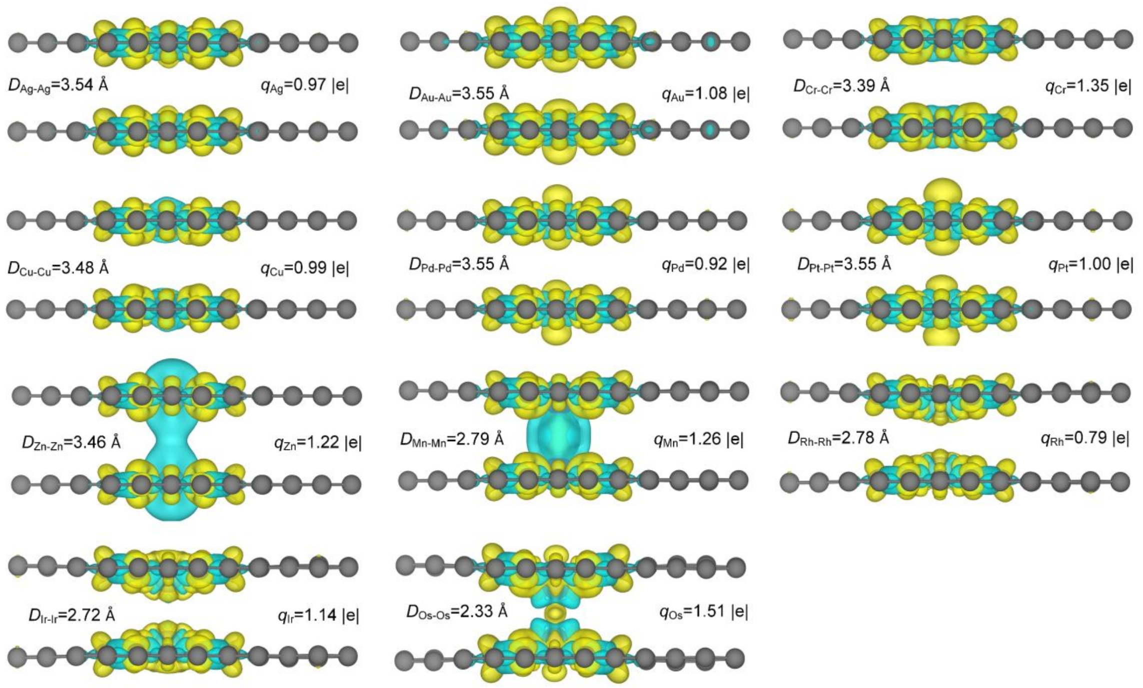

Figure S10. Charge density difference of MMC configurations. Yellow and cyan denote charge

accumulation and depletion, respectively. The isosurface value is set to 0.003 e Å^{-3}

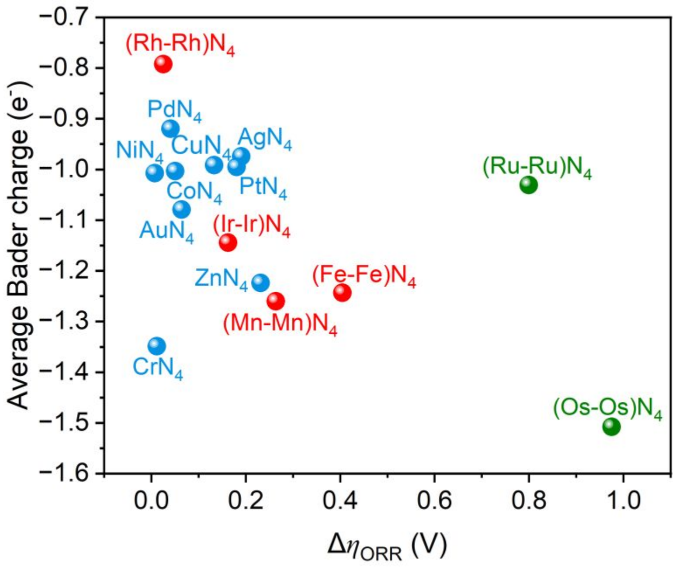

Figure S11. Correlation between the difference in predicted ORR limiting potential ($\Delta\eta_{ORR}$) by MMC and SL configurations with the calculated average Bader charge.

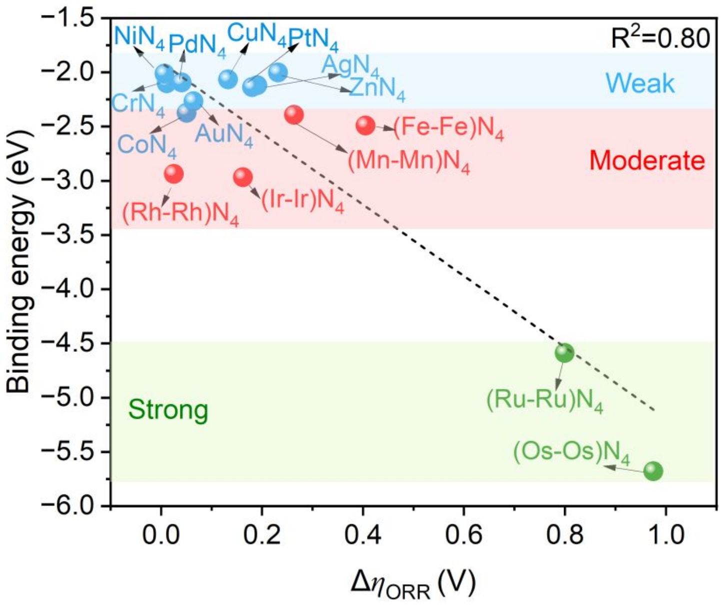

Figure S12. Correlation between the difference in predicted ORR limiting potential ($\Delta\eta_{ORR}$) by MMC and SL with the calculated binding energy.

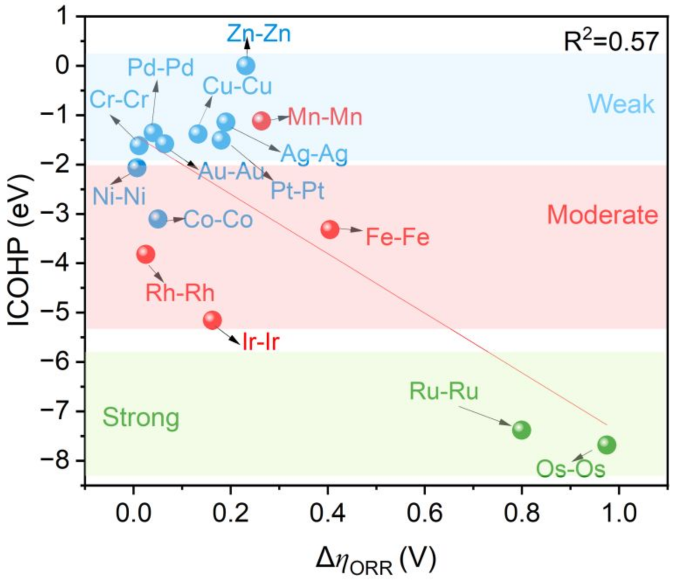

Figure S13. Correlation between the difference in predicted ORR limiting potential ($\Delta\eta_{ORR}$) and the ICOHP values calculated for isolated metal-metal dimers, used to probe intrinsic metal-metal interaction with the coordination environment removed.

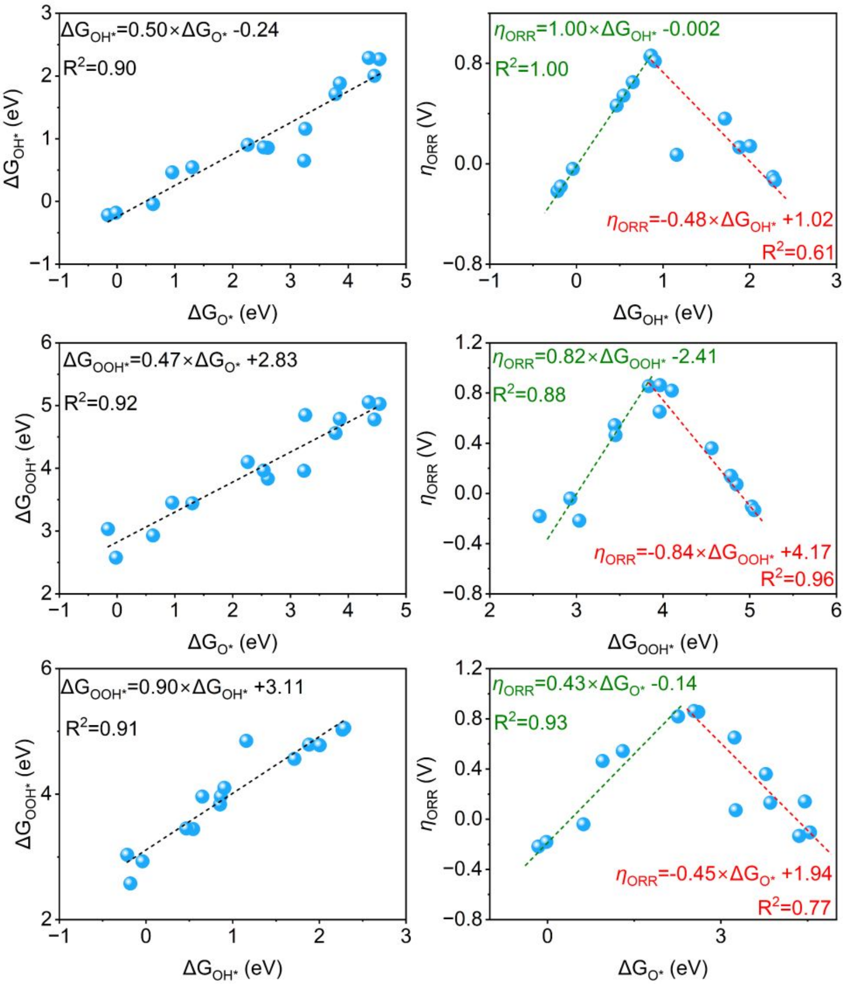

Figure S14. Relationships among intermediates in the mixed SL systems, and correlation between intermediates and η_{ORR}.

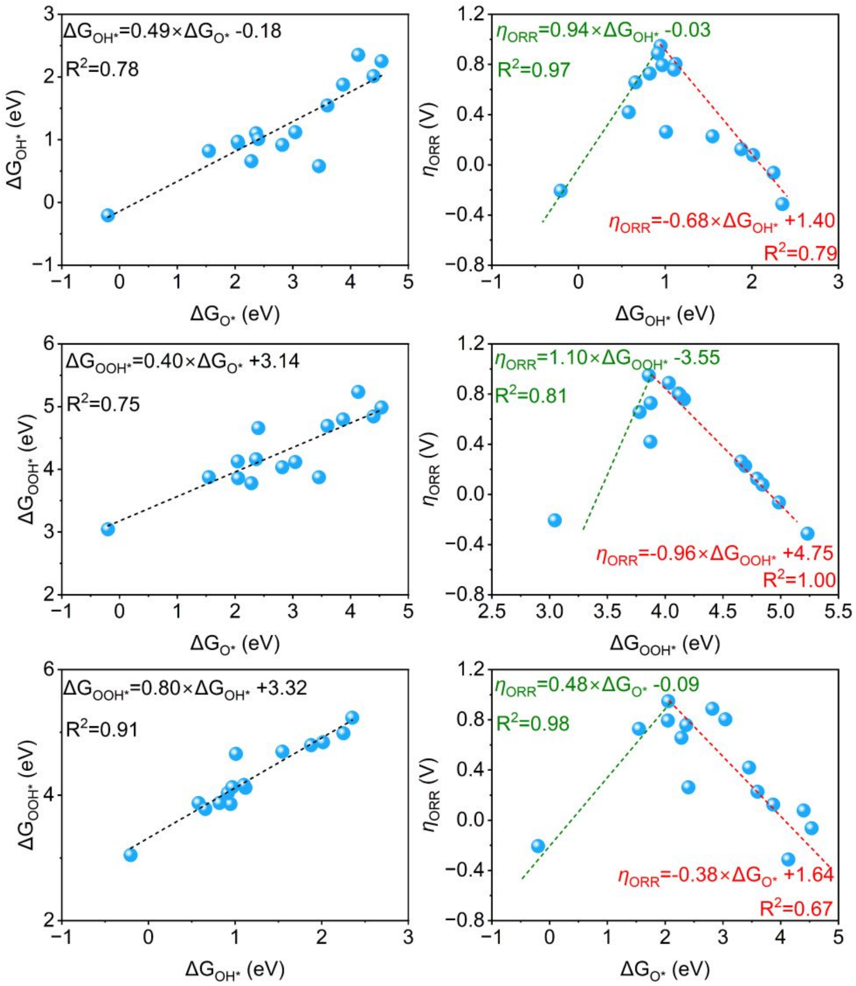

Figure S15. Relationships among intermediates in the mixed MMC systems, and correlation between intermediates and η_{ORR}.

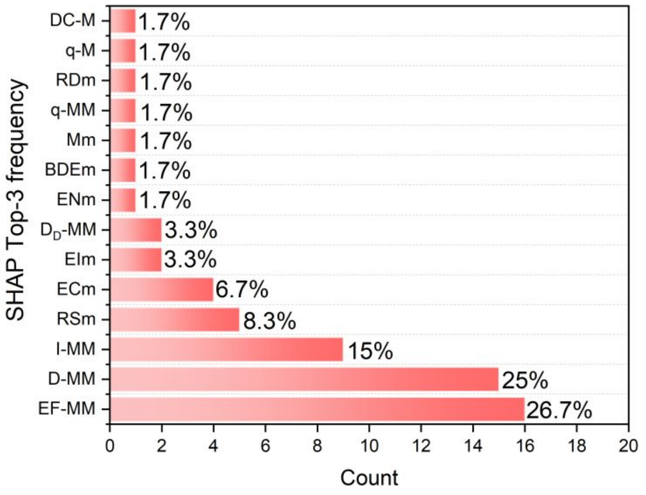

Figure S16. Resampling-based robustness of SHAP feature importance (Top-3 frequency).

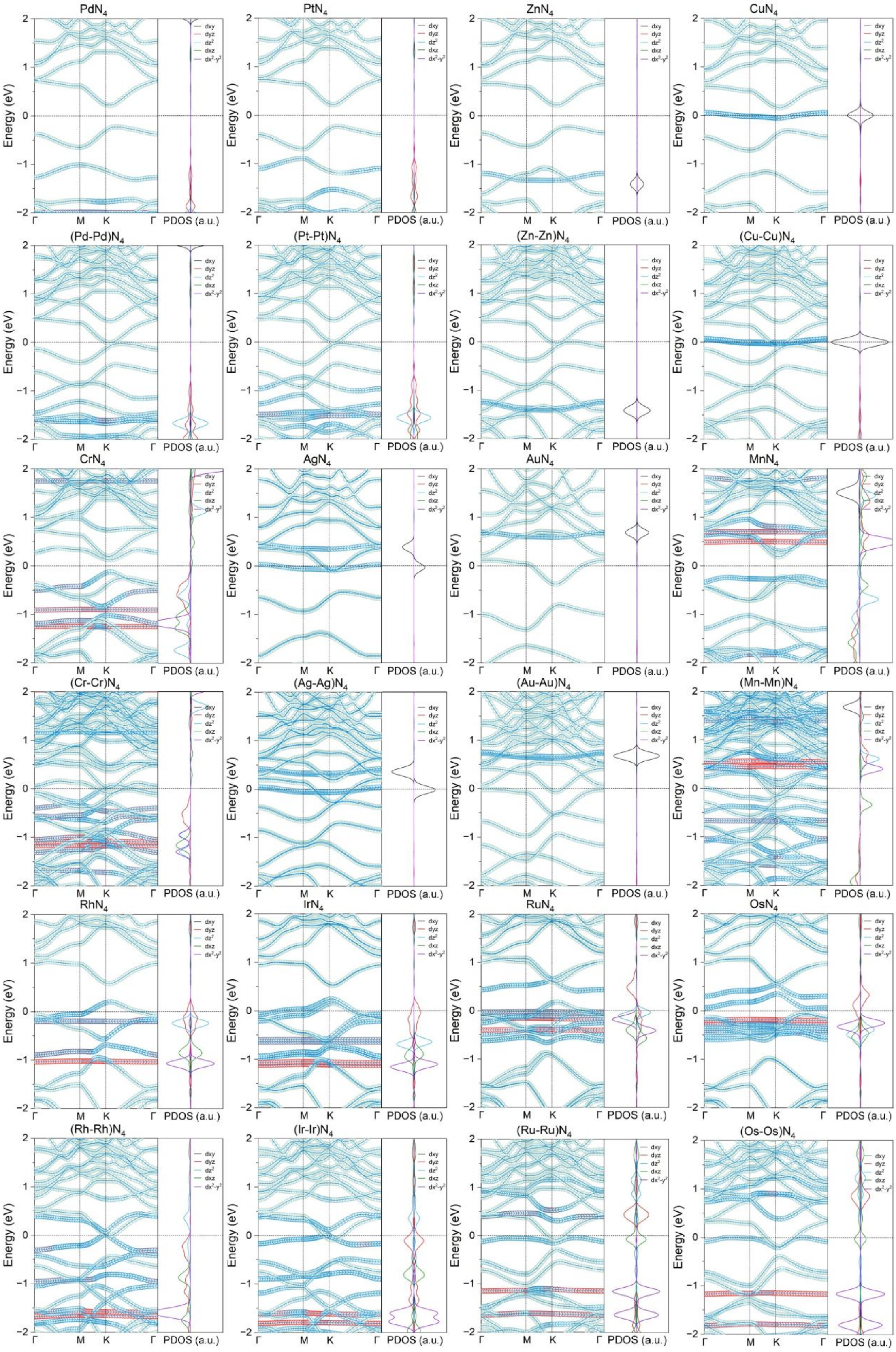

Figure S17. The projected band structure and projected density of states (PDOS) of transition metals for SL and MMC configurations.

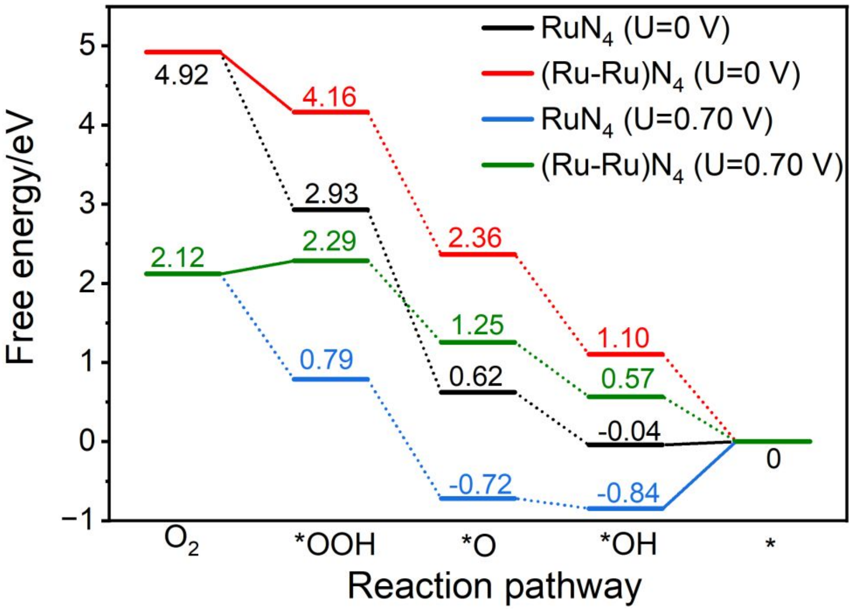

Figure S18. Reaction free energy profile for RuN_4 and $(Ru\text{-}Ru)N_4$ under the constant potential calculations.

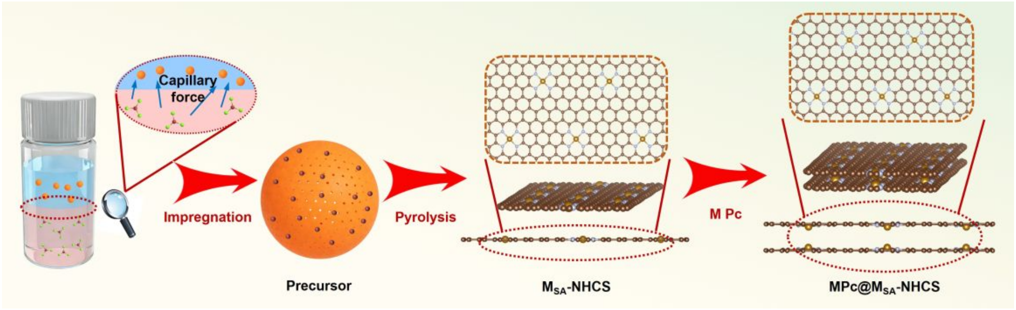

Figure S19. Schematic illustration of the process for the synthesis of MPc@M_{SA}-NHCS.

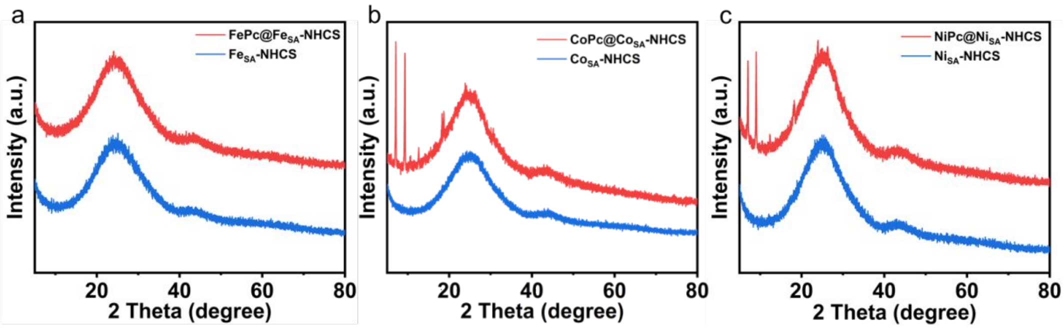

Figure S20. XRD patterns for (a) Fe-based catalyst, (b) Co-based catalyst and (c) Ni-based catalyst.

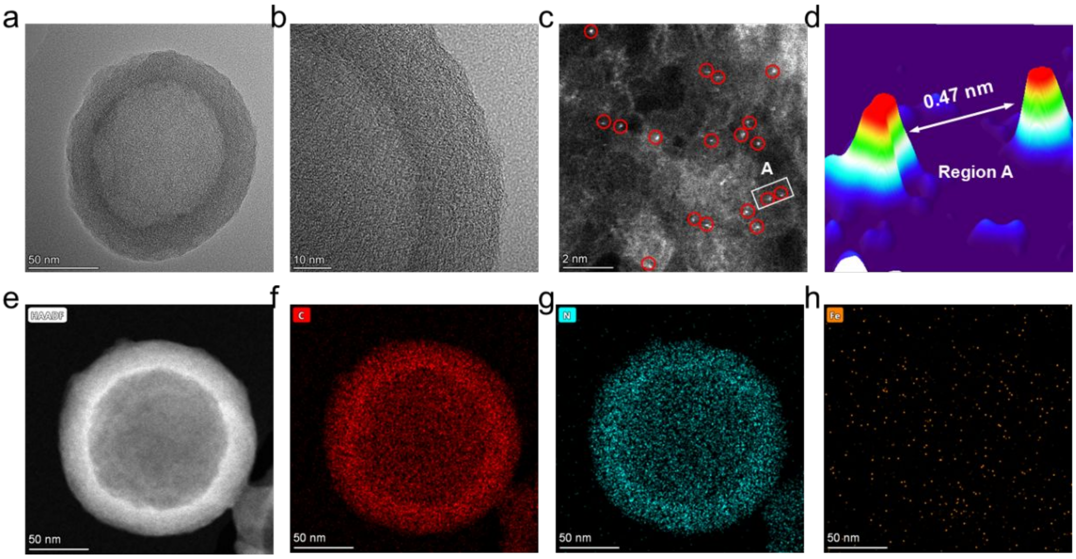

Figure S21. (a) TEM image, (b) HRTEM image and (c) Magnified AC HAADF-STEM images for Fe_{SA}-NHCS. (d) 3D atom-overlapping Gaussian-function fitting map of region A in (c) for the Fe_{SA}-NHCS. (e-h) HAADF-STEM image, and elemental mapping images for Fe_{SA}-NHCS.

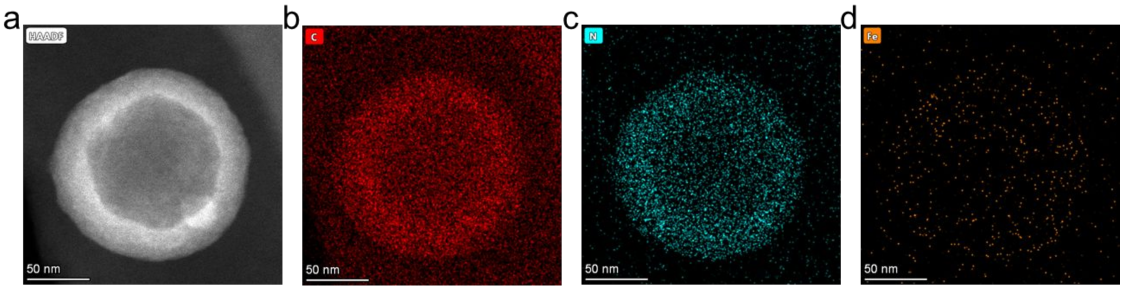

Figure S22. HAADF-STEM image, and elemental mapping images for FePc@Fe_{SA}-NHCS.

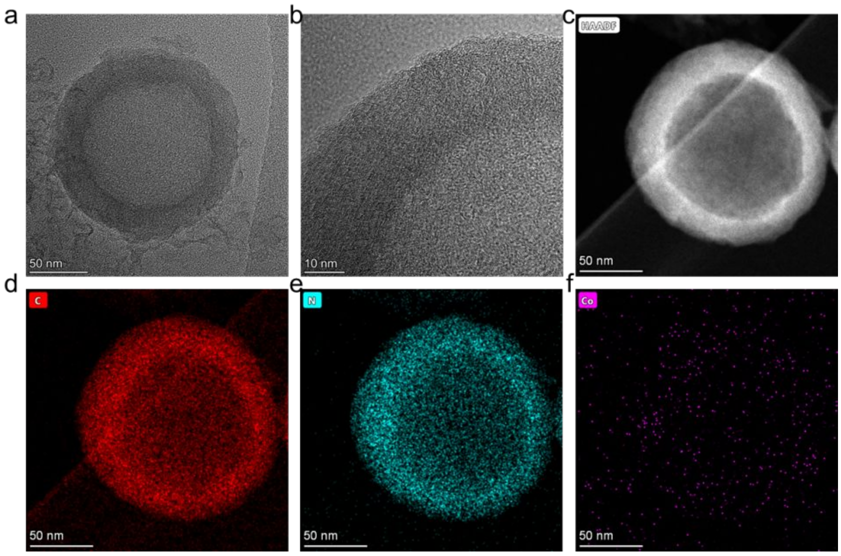

Figure S23. (a) TEM image, (b) HRTEM image, (c) HAADF-STEM image, and (d-f) elemental mapping images for Co_{SA}-NHCS.

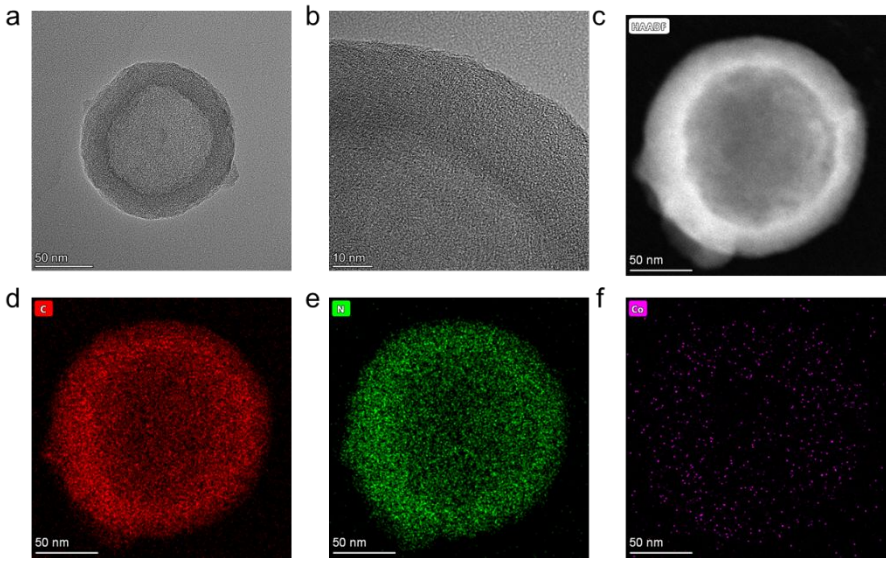

Figure S24. (a) TEM image, (b) HRTEM image, (c) HAADF–STEM image, and (d-f) elemental mapping images for CoPc@Co_{SA}-NHCS.

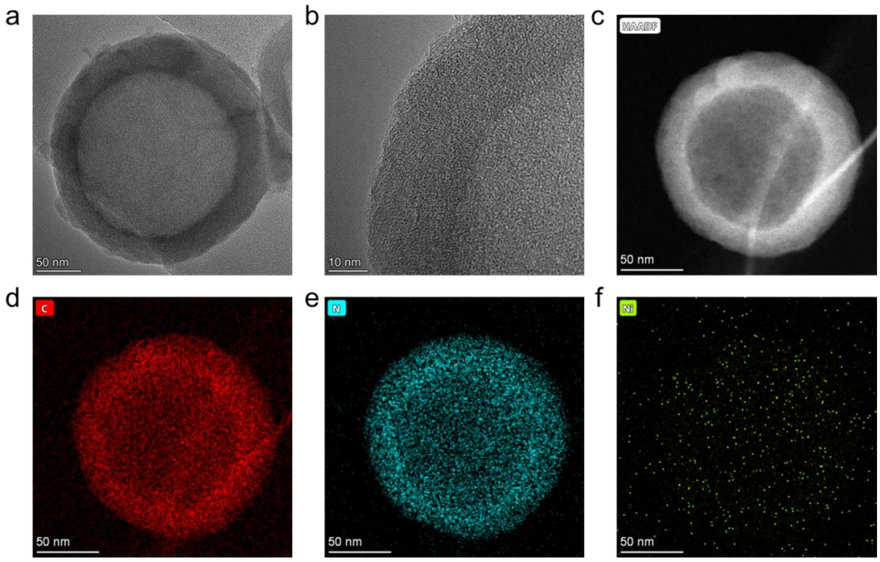

Figure S25. (a) TEM image, (b) HRTEM image, (c) HAADF–STEM image, and (d-f) elemental mapping images for Ni_{SA}-NHCS.

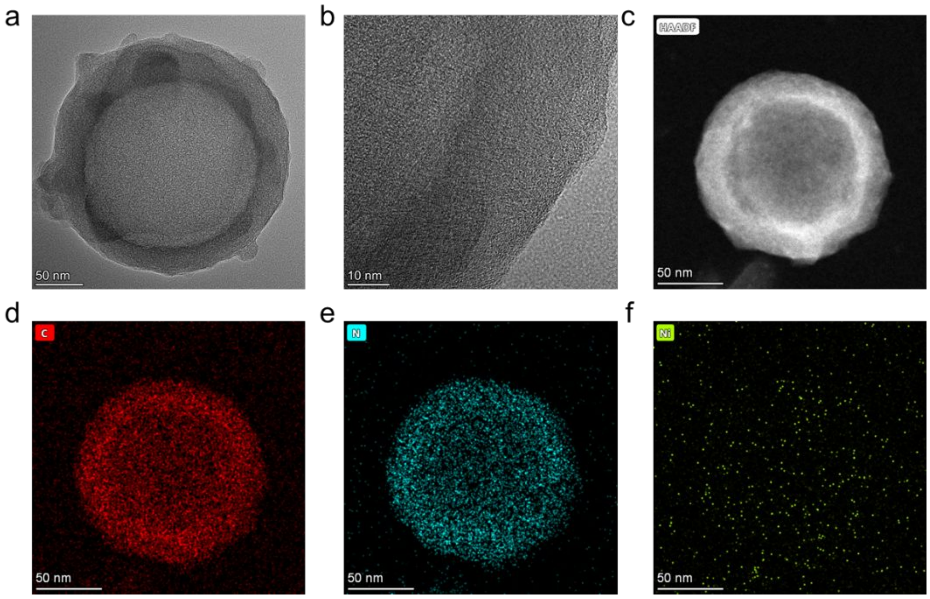

Figure S26. (a) TEM image, (b) HRTEM image, (c) HAADF–STEM image, and (d-f) elemental mapping images for NiPc@Ni_{SA}-NHCS.

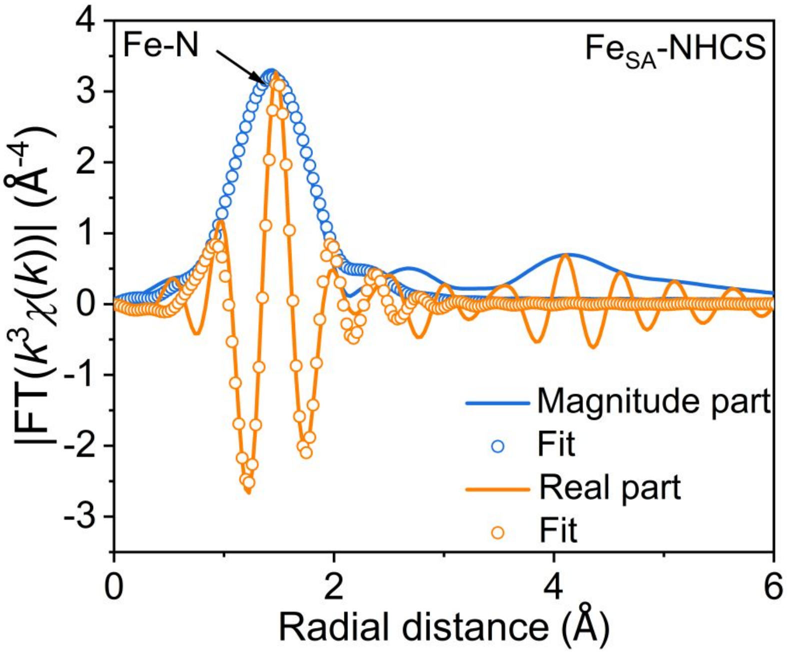

Figure S27. The corresponding k^3-weighted EXAFS fitting curves at R space for Fe_{SA}-NHCS.

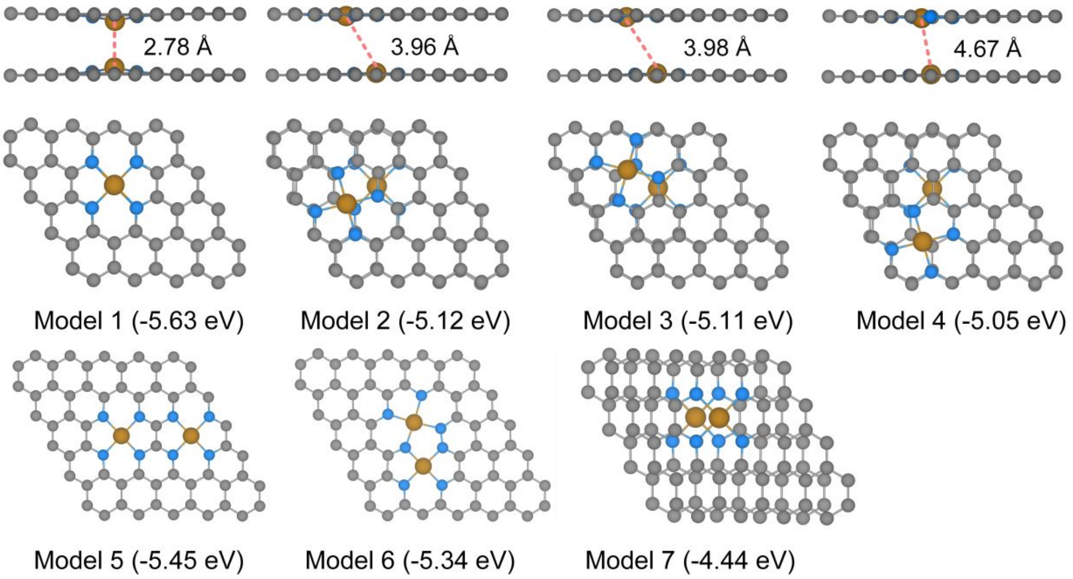

Figure S28. The possible dual atoms structures with their formation energies (Gold, blue, and gray spheres represent Fe, N, and C atoms, respectively).

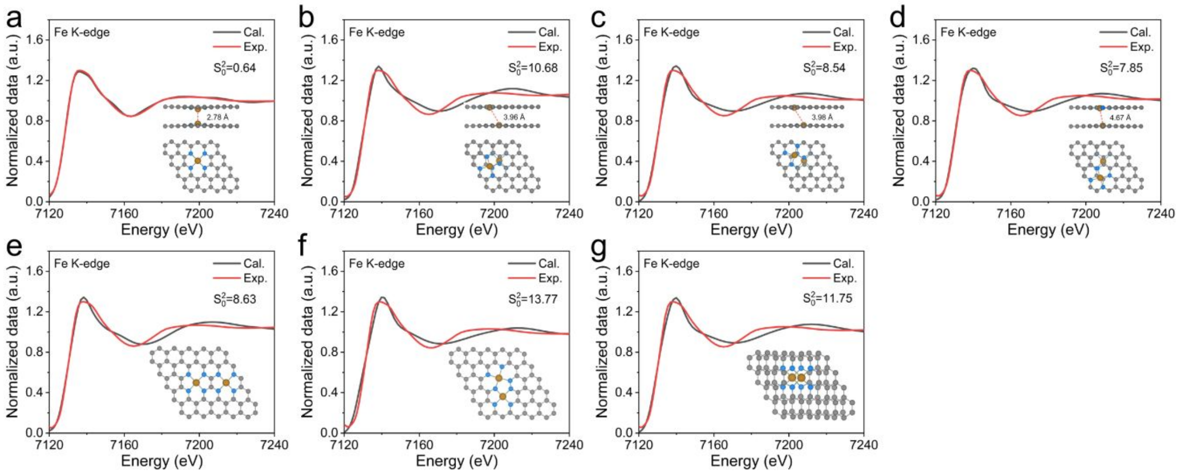

Figure S29. Comparison between the Fe K-edge XANES experimental spectrum of FePc@Fe_{SA}-NHCS (solid red line) and the theoretical spectrum (solid black line) calculated with different Fe atomic structures (including in plane dual sites models, Fe-Fe MMC models and laterally shifted bilayer registries).

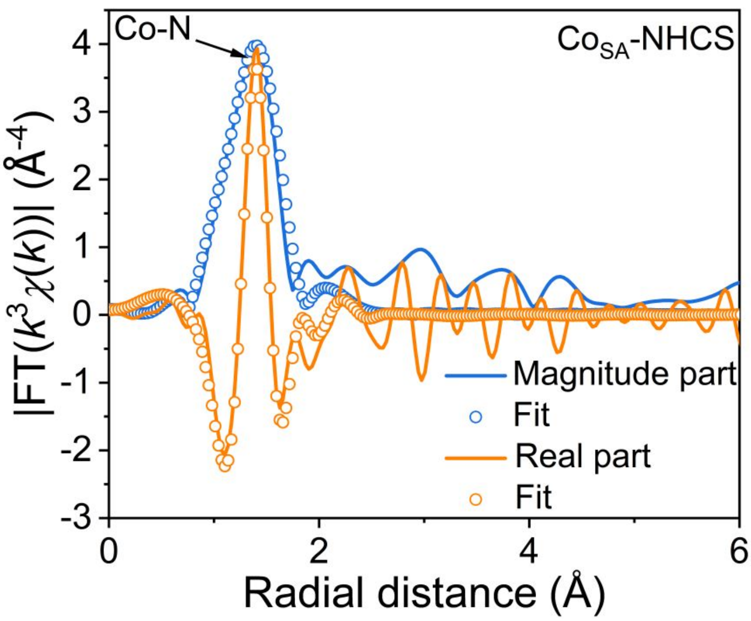

Figure S30. The corresponding k^3-weighted EXAFS fitting curves at R space for Co_{SA}-NHCS.

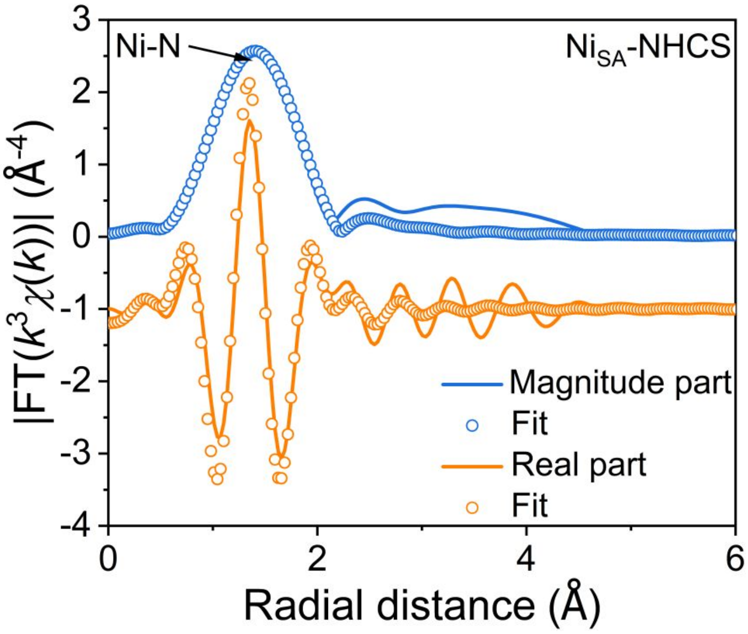

Figure S31. The corresponding k^3-weighted EXAFS fitting curves at R space for Ni_{SA}-NHCS.

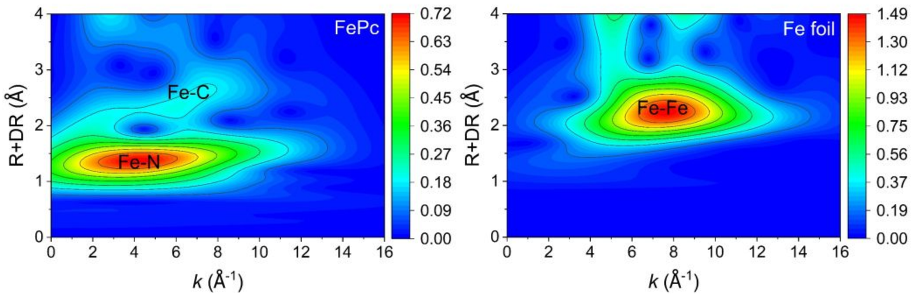

Figure S32. WT-EXAFS of FePc and Fe foil.

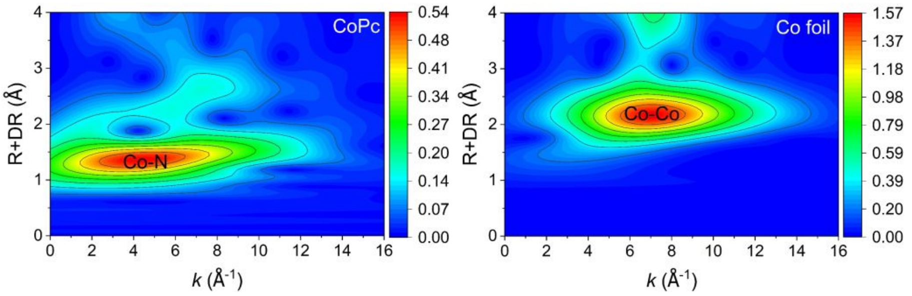

Figure S33. WT-EXAFS of CoPc and Co foil.

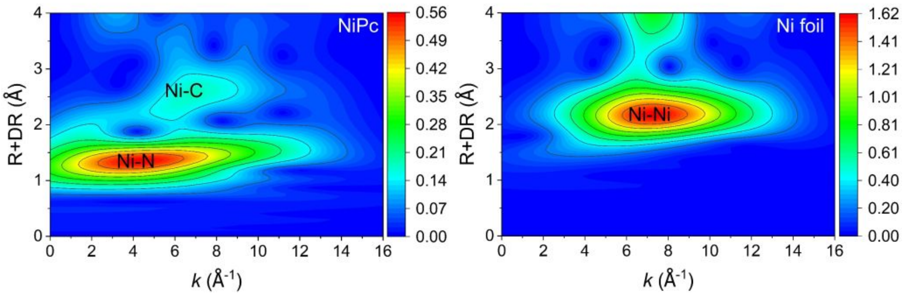

Figure S34. WT-EXAFS of NiPc and Ni foil.

Table S1. Comparison between calculated ORR limiting potentials and experimentally measured half-wave potentials.

Catalysts	Theoretical (V)	Experimental (V)	Ref.
FeN_4-HPC	0.47	0.76	*Energy Environ. Sci.,* **2022**, 15, 2619-2628
FeN_4	0.45	0.82	*ACS Catal.* **2021**, 11, 10, 6304–6315
FeN_4	0.47	0.79	*Angew. Chem. Int. Ed.* **2020**, 59, 13923 – 13928
FeN_4-0	0.6	0.82	*Adv. Mater.* **2023**, 35, 2300907
FeN_4	0.4	0.81	*Chem. Eng. J.* **2025**, 507, 160174
Pyridine-type FeN_4	0.56	0.71	*Energy Environ. Sci.*, **2020**, **13**, 111-118
Fe-SAs@PCF	0.631	0.856	*Adv. Funct. Mater.* **2025**, 35, 2418489
Fe_{SA} N-HCS	0.41	0.85	*Appl. Catal. B-Environ. Energy* **2025**, 365, 124990
Fe_{SA}@NMPC.	0.42	0.68	*Chem. Eng. J.* **2023**, 47, 146065
FeN_4@MCF	0.319	0.865	*Adv. Funct. Mater.* **2024**, 34, 2315150
FeN_4-NHC	0.4	0.79	*Angew. Chem. Int. Ed.* **2025**, 64, e202505937
Fe-NC	0.586	0.878	*Chem. Sci.*, **2024**, 15, 7259-7268
Fe SAs/N–C	0.47	0.798	*ACS Catal.* **2019**, 9, 2158–2163

Table S2. EXAFS data fitting results of Samples.

<table>
<tr><td>Sample</td><td>Path</td><td>CN[a]</td><td>R(Å)[b]</td><td>σ^2 (Å^2)[c]</td><td>ΔE_0(eV)[d]</td><td>R factor</td></tr>
<tr><td colspan="7">Co K-edge (S_0^2=0.756)</td></tr>
<tr><td>Co foil</td><td>Co-Co</td><td>12.0*</td><td>2.493</td><td>0.0063</td><td>8.7</td><td>0.0007</td></tr>
<tr><td rowspan="2">CoPc</td><td>Co-N</td><td>3.8</td><td>1.920</td><td>0.0027</td><td rowspan="2">10.4</td><td rowspan="2">0.0058</td></tr>
<tr><td>Co-C</td><td>7.1</td><td>2.943</td><td>0.0023</td></tr>
<tr><td>Co_{SA}-NHCS</td><td>Co-N</td><td>3.9</td><td>1.895</td><td>0.0118</td><td>-1.2</td><td>0.0127</td></tr>
<tr><td rowspan="2">CoPc@Co_{SA}-NHCS</td><td>Co-N</td><td>4.1</td><td>1.906</td><td>0.0029</td><td>4.0</td><td rowspan="2">0.0183</td></tr>
<tr><td>Co-Co</td><td>1.0</td><td>2.914</td><td>0.0086</td><td>5.0</td></tr>
<tr><td colspan="7">Ni K-edge (S_0^2=0.813)</td></tr>
<tr><td>Ni foil</td><td>Ni-Ni</td><td>12.0*</td><td>2.483</td><td>0.0061</td><td>8.5</td><td>0.0011</td></tr>
<tr><td rowspan="2">NiPc</td><td>Ni-N</td><td>4.3</td><td>1.903</td><td>0.0027</td><td>8.0</td><td rowspan="2">0.0102</td></tr>
<tr><td>Ni-C</td><td>7.6</td><td>2.949</td><td>0.0030</td><td>11.3</td></tr>
<tr><td>Ni_{SA}-NHCS</td><td>Ni-N</td><td>4.1</td><td>1.852</td><td>0.0040</td><td>0.7</td><td>0.0157</td></tr>
<tr><td rowspan="3">NiPc@Ni_{SA}-NHCS</td><td>Ni-N</td><td>3.9</td><td>1.885</td><td>0.0033</td><td>5.5</td><td rowspan="3">0.0095</td></tr>
<tr><td>Ni-C</td><td>6.5</td><td>2.916</td><td>0.0035</td><td>7.7</td></tr>
<tr><td>Ni-Ni</td><td>1.4</td><td>3.493</td><td>0.0051</td><td>-20.3</td></tr>
<tr><td colspan="7">Fe K-edge (S_0^2=0.730)</td></tr>
<tr><td rowspan="2">Fe foil</td><td>Fe-Fe</td><td>8.0*</td><td>2.465</td><td rowspan="2">0.0048</td><td rowspan="2">6.3</td><td rowspan="2">0.0058</td></tr>
<tr><td>Fe-Fe</td><td>6.0*</td><td>2.843</td></tr>
<tr><td rowspan="2">FePc</td><td>Fe-N</td><td>4.0</td><td>1.940</td><td>0.0051</td><td>9.6</td><td rowspan="2">0.0182</td></tr>
<tr><td>Fe-C</td><td>5.4</td><td>2.964</td><td>0.0031</td><td>10.1</td></tr>
<tr><td>Fe_{SA}-NHCS</td><td>Fe-N</td><td>3.8</td><td>1.882</td><td>0.0056</td><td>-10.9</td><td>0.0166</td></tr>
<tr><td rowspan="2">FePc@Fe_{SA}-NHCS</td><td>Fe-N</td><td>3.8</td><td>1.987</td><td>0.0131</td><td>-4.1</td><td rowspan="2">0.0058</td></tr>
<tr><td>Fe-Fe</td><td>1.0</td><td>2.827</td><td>0.0134</td><td>2.8</td></tr>
</table>

Table S3. The corresponding formation energies, S_0^2 of XANES spectra for the possible structures from model 1 to model 7.

**Model**	Formation energies (eV)	XANES S_0^2
**1**	-5.63	0.64
**2**	-5.12	10.68
**3**	-5.11	8.54
**4**	-5.05	7.85
**5**	-5.45	13.77
**6**	-5.34	11.75
**7**	-4.44	8.63

S_0^2 is the minimum of the square residue function in the space of the parameters, which is defined

as:

$$S_0{}^2 = n\frac{\sum_{i=1}^{m} W_i[(y_i{}^{th} - y_i{}^{exp})\varepsilon_i{}^{-1}]^2}{\sum_{i=1}^{m} W_i}$$

where n is the number of independent parameters, m the number of data points, y_i^{th} and y_i^{exp} the theoretical and experimental values of absorption, e^i the individual errors in the experimental data set, and w_i is a statistical weight.

References

1. Blöchl, P. E., Projector augmented-wave method. *Phys. Rev. B* **1994,** *50*, 17953.
2. Kresse, G.; Furthmüller, J., Efficient iterative schemes for ab initio total-energy calculations using a plane-wave basis set. *Phys. Rev. B* **1996,** *54*, 11169.
3. Kresse, G.; Joubert, D., From ultrasoft pseudopotentials to the projector augmented-wave method. *Phys. Rev. B* **1999,** *59*, 1758.
4. Grimme, S.; Antony, J.; Ehrlich, S.; Krieg, H., A consistent and accurate ab initio parametrization of density functional dispersion correction (DFT-D) for the 94 elements H-Pu. *J. Chem. Phys.* **2010,** *132*, 154104.
5. Aykol, M.; Wolverton, C., Local environment dependent GGA+ U method for accurate thermochemistry of transition metal compounds. *Phys. Rev. B* **2014,** *90*, 115105.
6. Garcia-Mota, M.; Bajdich, M.; Viswanathan, V.; Vojvodic, A.; Bell, A. T.; Nørskov, J. K., Importance of correlation in determining electrocatalytic oxygen evolution activity on cobalt oxides. *J. Phys. Chem. C* **2012,** *116*, 21077-21082.
7. Lany, S.; Raebiger, H.; Zunger, A., Magnetic interactions of Cr-Cr and Co-Co impurity pairs in ZnO within a band-gap corrected density functional approach. *Phys. Rev. B Condens. Matter Mater. Phys.* **2008,** *77*, 241201.
8. Xia, Z.; Xiao, H., Grand canonical ensemble modeling of electrochemical interfaces made simple. *J. Chem. Theory Comput.* **2023,** *19*, 5168-5175.
9. Choi, C.; Gu, G. H.; Noh, J.; Park, H. S.; Jung, Y., Understanding potential-dependent competition between electrocatalytic dinitrogen and proton reduction reactions. *Nat. Commun.* **2021,** *12*, 4353.
10. Trasatti, S., The absolute electrode potential: an explanatory note (Recommendations 1986). *Pure Appl. Chem.* **1986,** *58*, 955-966.
11. Cui, Y.; Ren, C.; Li, Q.; Ling, C.; Wang, J., Hybridization state transition under working conditions: activity origin of single-atom catalysts. *J. Am. Chem. Soc.* **2024,** *146*, 15640-15647.
12. Sutton, C.; Boley, M.; Ghiringhelli, L. M.; Rupp, M.; Vreeken, J.; Scheffler, M., Identifying domains of applicability of machine learning models for materials science. *Nat. Commun.* **2020,** *11*, 4428.
13. Kanungo, T.; Mount, D. M.; Netanyahu, N. S.; Piatko, C. D.; Silverman, R.; Wu, A. Y., An efficient k-means clustering algorithm: Analysis and implementation. *IEEE Trans. Pattern Anal. Mach. Intell.* **2002,** *24*, 881-892.
14. Han, Z.-K.; Sarker, D.; Ouyang, R.; Mazheika, A.; Gao, Y.; Levchenko, S. V., Single-atom alloy catalysts designed by first-principles calculations and artificial intelligence. *Nat. Commun.* **2021,** *12*, 1833.
15. Mazheika, A.; Wang, Y.-G.; Valero, R.; Viñes, F.; Illas, F.; Ghiringhelli, L. M.; Levchenko, S. V.; Scheffler, M., Artificial-intelligence-driven discovery of catalyst genes with application to CO2 activation on semiconductor oxides. *Nat. Commun.* **2022,** *13*, 419.
16. Goldsmith, B. R.; Boley, M.; Vreeken, J.; Scheffler, M.; Ghiringhelli, L. M., Uncovering structure-property relationships of materials by subgroup discovery. *New J. Phys.* **2017,** *19*, 013031.
